# Fine roots are the dominant source of recalcitrant plant litter in sugar maple‐dominated northern hardwood forests

**DOI:** 10.1111/nph.13494

**Published:** 2015-06-12

**Authors:** Mengxue Xia, Alan F. Talhelm, Kurt S. Pregitzer

**Affiliations:** ^1^ Department of Forest, Rangeland, and Fire SciencesUniversity of IdahoMoscowID83844‐1133USA

**Keywords:** acid‐insoluble fraction, chemical recalcitrance, fine roots, leaf litter, lignin, litter input, litter quality, nitrogen (N) deposition

## Abstract

- Most studies of forest litter dynamics examine the biochemical characteristics and decomposition of leaf litter, but fine roots are also a large source of litter in forests.
- We quantified the concentrations of eight biochemical fractions and nitrogen (N) in leaf litter and fine roots at four sugar maple (*Acer saccharum*)‐dominated hardwood forests in the north‐central United States. We combined these results with litter production data to estimate ecosystem biochemical fluxes to soil. We also compared how leaf litter and fine root biochemistry responded to long‐term simulated N deposition.
- Compared with leaf litter, fine roots contained 2.9‐fold higher acid‐insoluble fraction (AIF) and 2.3‐fold more condensed tannins; both are relatively difficult to decompose. Comparatively, leaf litter had greater quantities of more labile components: nonstructural carbohydrates, cellulose and soluble phenolics. At an ecosystem scale, fine roots contributed over two‐thirds of the fluxes of AIF and condensed tannins to soil. Fine root biochemistry was also less responsive than leaf litter to long‐term simulated N deposition.
- Fine roots were the dominant source of difficult‐to‐decompose plant carbon fractions entering the soil at our four study sites. Based on our synthesis of the literature, this pattern appears to be widespread in boreal and temperate forests.

## Introduction

Plant litter decomposition drives major flows of carbon (C) in soil systems: C mineralization and C storage (Chapin *et al*., 2011). As litter decomposes, C is returned to the atmosphere via respiration (Raich & Schlesinger, 1992); the remaining C provides a source of heterogeneous soil organic C that accounts for approximately two‐thirds of terrestrial C (Batjes, 1996). Knowledge of plant litter decomposition and its controlling factors are foundational elements of terrestrial biogeochemical models used to understand the effects of global change on C and nutrient cycling (McGuire *et al*., 2001).

Plant litter is derived from various plant organs, such as leaves (Aber & Melillo, 1982), roots (Gill & Jackson, 2000) and woody stems (Dearden *et al*., 2006). Of these organs, decomposition research has primarily focused on leaf litter, likely because leaf litter is a large and visible input to the soil that can be easily sampled. Consequently, leaf litter decomposition processes, including the rate, chemistry and biology of decomposition, are often assumed to be broadly representative of plant litter decomposition (Rasse *et al*., 2005; Freschet *et al*., 2013). This assumption has been used extensively in models of ecosystem C cycling (Schmidt *et al*., 2011).

However, growing evidence suggests that our knowledge of leaf decomposition may be inadequate for the purpose of broadly understanding plant litter dynamics. First, it is now apparent that fine roots contribute a substantial portion of plant litter production. In a recent meta‐analysis of litter inputs in tropical, temperate and boreal/alpine forests, root litter accounted for 48% of annual plant litter inputs, greater than either leaf litter (41%) or fine stems (11%; Freschet *et al*., 2013). In some forests, root litter was estimated to contribute over two‐thirds of the litter production (Grier *et al*., 1981). Second, evidence from litterbag and isotopic tracer studies has demonstrated that fine roots generally break down more slowly than leaves. Litter bag studies have shown that root litter decays slower than leaf litter across a range of terrestrial ecosystems, from boreal forests to sub‐humid savannas (Taylor *et al*., 1991; Lehmann *et al*., 1995; Gholz *et al*., 2000; Abiven *et al*., 2005; Kelleher *et al*., 2006; Vivanco & Austin, 2006; Freschet *et al*., 2013 *;* van Huysen *et al*., 2013 *;* but see Ostertag & Hobbie, 1999). At the end of a decade‐long litterbag experiment that used nine litter types and 27 sites across North and Central America, approximately one‐third more root litter than leaf litter remained (estimated from Harmon *et al*., 2009). In isotopic tracer studies, fine root inputs resulted in approximately one‐third more soil C retention than leaf litter in a temperate forest (Bird & Torn, 2006; Bird *et al*., 2008) and an acidic tundra soil (Loya *et al*., 2004). In temperate deciduous forest soils, isotopic measurements estimated that root‐derived C represented > 60% of microbial biomass after 3 yr of litter addition (Kramer *et al*., 2010). Rasse *et al*. (2005) found that roots contributed an average of two‐fold more to soil organic C than leaf litter in a summary of agricultural studies using isotope tracer techniques. More recent research using pyrolysis and compound‐specific isotope analysis showed that fine root‐derived C was more abundant than leaf‐derived C in the humin fraction, a recalcitrant part of soil organic matter (Mambelli *et al*., 2011). Taken together, these lines of evidence demonstrate a need for biogeochemical models to incorporate more experimental data on fine root chemistry and decomposition dynamics.

It is not clear why fine roots are generally more resistant to decomposition than leaf litter. Decomposition is controlled by both exogenous factors, such as environmental conditions (Zhang *et al*., 2008; Solly *et al*., 2014), decomposer community composition (Wickings *et al*., 2012) and interactions with soil particles (Six *et al*., 2002), and endogenous factors, such as tissue chemistry (Melillo *et al*., 1982; Adair *et al*., 2008). Because fine roots and leaves are initially added to the soil at different locations, exogenous factors likely account for some of the differences in decomposition (Rasse *et al*., 2005). However, a number of experiments have observed that fine roots degrade slower than leaf litter when exogenous factors were held constant (i.e. both types were put in the same soil depth at the same locations, Taylor *et al*., 1991; Abiven *et al*., 2005; Bird *et al*., 2008), and such a pattern persists even when fine roots were milled to fine particles before incubation in soil (Waid, 1974). These results suggest that fine roots are more chemically‐resistant to decomposition, which could be one of the mechanisms that contribute to the greater retention of root‐derived C within soil. Consequently, we hypothesized that fine roots are more biochemically‐resistant to decomposition than leaf litter and that fine roots are the dominant source of recalcitrant plant materials returned to soil.

We tested this hypothesis by investigating the biochemistry of leaf litter and fine roots at four sugar maple‐dominated hardwood forests across a 500‐km climate and air pollution gradient and by modeling inputs of specific classes of compounds to soil. We quantified the concentrations of nitrogen (N) and eight major plant biochemical fractions/classes for both tissue types. Here, we refer to a plant tissue as ‘recalcitrant’ if it contains high concentrations of major biochemical fractions/classes resistant to microbial degradation and reported to retard litter decomposition, such as the acid‐insoluble fraction (AIF, conventionally referred to as lignin; Melillo *et al*., 1982; Taylor *et al*., 1989; Sariyildiz & Anderson, 2003; Cornwell *et al*., 2008; Amin *et al*., 2014) and condensed tannins (Wardle *et al*., 2002; Hättenschwiler *et al*., 2011). Likewise, recalcitrant tissues also have relatively low concentrations of easily degraded substrates, such as nonstructural carbohydrates (Waldrop & Firestone, 2004) and simple phenolics (Hättenschwiler *et al*., 2011). We also calculated litter quality indices including the ratios of C and N (C : N), AIF and N (AIF : N), and AIF and the sum of AIF + holocellulose (lignocellulose index). These indices have been reported to be negatively correlated with decomposition rates across a large number of ecosystems (Taylor *et al*., 1989; Preston *et al*., 2000; Adair *et al*., 2008; Zhang *et al*., 2008). Our assessment of chemical recalcitrance was primarily based on C quality and largely neglected the role of mineral nutrient availability on litter decomposition (Paul, 2006). Mineral nutrient availability is less likely to influence decomposition on relatively fertile sites (Melillo *et al*., 1982), such as those used in this study (Pregitzer *et al*., 2008). Because forest productivity, root biomass and root turnover at these sites are well‐documented (Burton *et al*., 2000; Pregitzer *et al*., 2008), we were also able to estimate the relative contribution of leaves and fine roots to the total flux of recalcitrant and labile compounds returned to soil.

Because fine roots and leaves have different physiological functions, our second aim was to investigate if leaf litter and fine roots have unique biochemical responses to environmental change. For example, experimental soil warming decreased the amount of added ^15^N that was allocated to old leaves, but increased the ^15^N recovery in fine roots (Hobbie & Chapin, 1998). Thus, the response of leaf litter to environmental change may not represent the overall shift in litter chemistry. To compare how fine root and leaf litter biochemistry respond to environmental change, we took advantage of our long‐term simulated N deposition experiment. We have already documented a number of responses to simulated N deposition at these sites, including increased soil organic C (Pregitzer *et al*., 2008), an effect explained by slower litter decomposition (Zak *et al*., 2008). Also, simulated N deposition significantly increased canopy leaf N concentration, but did not affect fine root N concentration (Zak *et al*., 2008), suggesting that leaf litter and fine root chemistry have responded differently to simulated N deposition. Accordingly, we hypothesized that long‐term simulated N deposition alters litter biochemistry to favor slower decomposition and that the biochemistry of leaf litter and fine roots responds differently to long‐term simulated N deposition at these study sites.

## Materials and Methods

### Site description

The four study sites encompass the north–south distribution of the northern hardwood forest biome in the Great Lakes region of North America and occur along a 500‐km temperature and N deposition gradient (Table 1). These sites are heavily dominated by sugar maple (*Acer saccharum* M.) and similar in stand composition, age and soil properties (Table 1). The O_e/a_ horizon at these sites is permeated by a dense mat of sugar maple fine roots and contains a large amount of C (Zak *et al*., 2008; Table 1). Soils are sandy (Kalkaska series, Typic haplorthod) and pH values range from 4.4 to 4.7 in the top 10 cm of mineral soil. Six 30‐m × 30‐m plots were established at each site, each plot surrounded on all sides by a 10‐m buffer treated the same way as the main plot area. Since 1994, three plots at each site have received experimental additions of N at a rate (3 g N m^−2^ yr^−1^ as NaNO_3_ in six equal increments across the growing season) similar to rates of N deposition occurring in some areas of Europe (Holland *et al*., 2005).

**Table 1:** Location, climate and edaphic characteristics of the four northern hardwood forest study sites

Site characteristic	Site A	Site B	Site C	Site D
Latitude (N)	46°52′	45°33′	44°23′	43°40′
Longitude (W)	88°53′	84°51′	85°50′	86°09′
Mean annual precipitation (mm)^a^	873	871	888	812
Mean annual temperature (°C)^a^	4.7	6.0	6.9	7.6
Ambient wet + dry N deposition (g N m^−2^ yr^−1^)^b^	0.68	0.91	1.17	1.18
Growing season length (d)^a^	134	150	154	157
Total basal area (m^2^ ha^−1^)^c^	34	31	32	33
Sugar maple basal area (%)^c^	86	86	83	75
Overstory age (2015)	108	102	103	107
Ambient soil carbon content (0–70 cm, g m^−2^)^a^	8341	9259	7841	7470
O_e+a_	350	625	720	640
0–10 cm soil depth	2427	3126	2560	2113
Soil texture, 0–10 cm depth (% sand‐% silt‐%clay)^a^	75‐22‐3	89‐9‐2	89‐9‐2	87‐10‐3
Soil texture, 10–70 cm depth (%sand‐% silt‐% clay)^a^	84‐11‐5	88‐7‐5	91‐6‐3	92‐5‐3

a Pregitzer *et al*. (2008).

b MacDonald *et al*. (1992)

c Burton *et al*. (2000).

### Leaf litter and fine root sampling

Sugar maple leaf litter was collected in litter traps randomly located in each plot in the autumn of 2010 following the protocol of Pregitzer *et al*. (2008). Root mortality is relatively evenly distributed over the growing season at these sites (Burton *et al*., 2000), so we collected fine roots at two points in the growing season – October 2010 (autumn) and May 2011 (spring) – to better describe the chemical characteristics of fine roots throughout the growing season. We used the mean of spring and autumn fine roots to represent fine roots unless otherwise stated. At six to eight random points within the buffer of each plot, we removed the O_i_ and excavated fine roots from the top 10 cm of soil, including the O_e+a_ horizon, A horizon and a portion of the E horizon. Roots were sorted by hand and identified to the genus *Acer* by morphological characteristics. Maples other than the sugar maple contributed only an average of 7.5% to stand basal area in 2011. Young roots, visually identified as white and turgid, were removed to minimize the difference between the root tissue we sampled and root necromass. Because we are also conducting a decomposition study, the number of samples varied in order to collect *c*. 300 g of fine roots per plot. We assumed that the chemical qualities of the leaf litter and fine roots we sampled represented the forest as a whole, because sugar maple represents 77% of the annual leaf litter flux at these sites and the genus *Acer*, whose fine roots we sampled, contributes 90% of overstory basal area and 83% of all woody groundcover stems (Talhelm *et al*., 2013).

Following initial processing to quickly remove mineral soil, organic matter and roots of other species, the root samples of each plot were rinsed, homogenized and flash frozen in liquid N_2_. They were then packed with solid CO_2_ for shipping to the University of Idaho, where we isolated first‐ to third‐order roots for analysis (with the most distal root‐tips defined as first order; Pregitzer *et al*., 2002). We used the first‐ to third‐order roots because these roots represent the most short‐lived and metabolically active portion of the root system (Guo *et al*., 2008; Valenzuela‐Estrada *et al*., 2008; Xia *et al*., 2010; McCormack *et al*., 2015), better serving as the belowground counterpart to foliage (Li *et al*., 2010a). In comparison, when the fine roots of trees are defined as those < 2 mm in diameter, this pool includes a large number of roots that are longer‐lived and tend to undergo secondary thickening (Xia *et al*., 2010; McCormack *et al*., 2015). Further, the first three order roots we sampled had mean diameters *c*. 0.30 mm (Supporting Information Table S1), similar to the mean diameter observed in minirhizotrons at our sites (*c*. 0.31 mm, Burton *et al*., 2000). Approximately 2 g DW of roots for each plot were used for the chemical analyses presented here.

### Substrate biochemistry

We analyzed plant tissues for total C and N, nonstructural carbohydrates (NSCs), soluble phenolics, condensed tannins (CTs), soluble proteins, total lipids, AIF and hemicellulose. Total C and N were analyzed with an elemental analyzer (ECS 4010, Costech Analytical, Valencia, CA, USA). For NSCs, samples were extracted with 80% ethanol and analyzed for sugars using phenol‐sulfuric acid (Chow & Landhäusser, 2004). The residues were digested with a mixture of α‐amylase/amyloglucosidase for starch determination. Starch‐digesting enzymes exhibit more complete starch digestion than acid hydrolysis and lack the capability to inadvertently degrade structural polysaccharides (Chow & Landhäusser, 2004). After digestion, the glucose hydrolyzates were measured colorimetrically with a peroxidase‐glucose oxidase/o‐dianisidine reagent (Chow & Landhäusser, 2004). Soluble phenolics were extracted with 70% acetone and determined with Folin‐Ciocalteu (FC) reagent as catechin equivalents (Booker *et al*., 1996). This protocol quantifies phenolics as the overall capacity to reduce heteropolyphosphotungstates‐molybdates to blue complexes (Singleton *et al*., 1999); other reducing reagents that also react with FC reagent such as ascorbic acids and aromatic amino acids may also contribute to this reducing capacity. However, the occurrence of these compounds in mature leaves and fine roots are minor (mostly < 0.05%; Cyr *et al*., 1990; Tschaplinski *et al*., 1995; Chávez *et al*., 2000; Chen & Gallie, 2005), and further decreased in senesced litter (Buchanan‐Wollaston, 1997; Gergoff *et al*., 2010). The extractable CTs were extracted with repeated sonication in 70% acetone (Yu & Dahlgren, 2000) and determined by acid‐butanol assay (Booker *et al*., 1996). We prepared CT standards from apple fruits following the protocols of Li *et al*. (2010b). Because there is no generally accepted CT standard and different CT structures can react differently to the assay, the CT quantification in this study should be interpreted as a relative assessment of CT concentrations rather than an absolute quantification. However, Coq *et al*. (2010) observed a strong correlation between the acid butanol assay and HPLC quantification of CTs in 15 species of leaf litter (*r *=* *0.934). The insoluble residues were freeze‐dried, re‐suspended in methanol, incubated at 95°C and determined for bound CTs (Booker *et al*., 1996). Total CTs were the sum of extractable and bound fractions. Soluble proteins were extracted with 0.1 M NaOH and determined by Coomassie Protein Bradford Reagent (Thermo Fisher Scientific Inc., Rockford, IL, USA) with the addition of diluted polyvinylpyrollidone (Fisher BioReagent, Pittsburgh, PA, USA) to minimize the interference by brown quinones (Jones *et al*., 1989). Bovine serum albumin (Thermo Fisher Scientific) was used to construct the standard curve. For total lipids, samples were homogenized using methanol/chloroform/water, adapted from Bligh & Dyer (1959). Water was added to the supernatants, which then separated into two layers. Lipid contents in the chloroform layer were determined gravimetrically after evaporating chloroform to dryness (Smedes & Thomasen, 1996).

Extractive‐free fraction, referred to as cell‐wall fraction in this study, was prepared by processing samples with sequential extractions (Methods S1). These washes removed both polar and nonpolar extractives that are considered readily decomposable, leaving highly cross‐linked cell wall components in the residues (Aber *et al*., 1990; Hendricks *et al*., 2000). The total of polar and nonpolar extractives in this study is referred to as the ‘extractive fraction’. The remaining residues were subsequently dried and weighed to determine the cell‐wall fraction. The extractive fraction is the difference between initial weight and the weight of the cell‐wall fraction. The cell‐wall fraction then was divided into acid‐soluble and acid‐insoluble fractions (AIF) using a two‐phase H_2_SO_4_ hydrolysis adapted from Booker *et al*. (1996). The acid‐soluble fraction, consisting of dominantly cell‐wall polysaccharides, along with other compounds, for example phenolics and lipids, linked to cell wall via ester bonds (Iiyama *et al*., 1994; Preston *et al*., 2000), was hydrolyzed by H_2_SO_4_ incubation_._ The remaining residues were dried and weighed to determine the AIF. For hemicellulose, the pellets remaining after tannins extraction were incubated with 10% KOH for 24 h at 30°C (Dickson, 1979; Chapman *et al*., 2005). The extracts were mixed with 4 M acetic acid in ice‐cold ethanol for 24 h. The precipitate was dried to determine hemicellulose concentration. Cellulose was calculated as the cell‐wall fraction concentration minus the concentration of AIF and hemicellulose. Lignocellulose index was the ratio of AIF to cell‐wall fraction. Ash contents were determined after 4 h combustion in a muffle furnace at 500°C: these were 7.6 ± 1.3% in leaf litter and 3.6 ± 1.3% in fine roots. All concentrations were expressed on an ash‐free dry mass basis.

### Annual litter flux

The annual fluxes of biochemical classes to soil through leaf litter or fine roots were calculated as:

$$I_{a}=P_{l}\times C_{a}$$

(*I*_*a*_, annual input of an individual biochemical class; *P*_*l*_, annual litter production of leaf litter or fine roots; *C*_*a*_, concentration of the biochemical class in leaf litter or fine roots).

The annual leaf litter production was estimated from the leaf litter trap collections in each plot (Pregitzer *et al*., 2008) and was averaged for each plot from annual measurements of 1988–2011 for ambient plots, and 1994–2011 for N‐amended plots (data available at Michigan Nitrogen Deposition Gradient Study database, http://webpages.uidaho.edu/nitrogen-gradient). Leaf litter production and fine root biomass at these sites have changed little through time (Talhelm *et al*., 2012) or as a result of simulated N deposition (Burton *et al*., 2004; Pregitzer *et al*., 2008). Similarly, simulated N deposition has not affected fine root turnover (Burton *et al*., 2004). In the year that we sampled the leaf litter for biochemistry (2010), leaf litter production was within ± 10% of the long‐term average.

The annual litter production of fine roots included roots in the top 70 cm of the soil and was estimated as the standing biomass of fine roots within a specific soil depth increment multiplied by the corresponding fine root turnover rate for that soil increment for each plot. The fine root turnover rate at each soil depth in each plot was derived from minirhizotron observations at these sites (Burton *et al*., 2000). Fine root biomass data for 0–10 cm, 10–30 cm, 30–50 cm and 50–70 cm in soil depth were obtained by soil cores at each plot in 2004 and 2009 (data available at Michigan Nitrogen Deposition Gradient Study database), which classified roots by diameter, rather than branch order. We used the data from the smallest diameter class (< 0.5 mm) in these surveys. We believe that the roots we sampled for biochemical analyses (the first three root orders) are analogous to those included in the estimates of root litter production because nearly all roots (*c*. 97%) among the small root branches of sugar maple are found within the first three root orders (Pregitzer *et al*., 2002). Further, there is good correspondence between the mean diameter of the roots observed via minirhizotron (0.31 mm; Burton *et al*., 2000) and the diameter of the three root orders we sampled (*c*. 0.30 mm, Table S1). However, we are aware that the chemical traits of fine roots collected from the top 10 cm of soil may differ somewhat from those deeper in the soil. The influence of these differences on biochemical fluxes should be limited. Fine root production and turnover decrease with depth in temperate hardwood forests (Joslin *et al*., 2006); fine roots within the top 10 cm of soil represent 52% of the fine root biomass within the top 70 cm of soil and 72% of the root turnover in the top 50 cm of soil at our sites (Burton *et al*., 2000). Further, this study predominantly focused on major C fractions rather than element concentrations, such as phosphorus, sodium, and potassium that vary strongly by soil depth.

### Statistical analysis

We tested whether the biochemical traits and fluxes differ among tissue types (leaf litter vs fine roots, or spring vs autumn roots, df = 1), simulated N deposition (df = 1) and study sites (df = 3) using a split‐plot design analyzed with mixed linear models (Proc Mixed, Littell *et al*., 2006), followed by Tukey's HSD tests for pairwise comparisons. In this model, sites, N treatments and their interactions were sources of whole‐plot variation, whereas tissue type was the within‐plot factor. The interaction terms of tissue type and treatment tested the hypothesis whether different tissue types responded differently to simulated N deposition. We also used a two‐way ANOVA to determine whether simulated N deposition (df = 1), site (df = 3) or their interactions (df = 3) had effects on the leaf litter production, fine root mass turnover, total litter production, and the sum of leaf litter and fine root fluxes of each biochemical class. Data were log‐transformed before being analyzed in SAS 9.3 (SAS Institute Inc., Cary, NC, USA) to reduce the effects of variations increasing with means.

## Results

### Biochemical composition and nitrogen

We investigated the abundance of eight major biochemical fractions (representing *c*. 90% of substrate dry mass; Table 2), N concentration and three litter quality indices for leaf litter and fine roots collected from four sugar maple‐dominated hardwood forests in the north‐central United States. Tissue type (leaf litter vs fine roots) resulted in a considerably greater variance than both site and simulated N deposition for all biochemical traits (Tables 2, S2, S3). Leaf litter had substantially higher concentrations of nonstructural carbohydrates (NSCs) and lipids (*P* < 0.001, Table 2). Leaf litter also exhibited higher concentrations of cellulose and soluble phenolics than fine roots in general (Table 2), but the magnitude of difference varied among sites (tissue × site, *P* < 0.05, Tables S2, S3). The amount of unidentified material was generally higher in leaf litter than in fine roots, a trend that was strongest at site C (tissue × site: *P *=* *0.013, Tables 2, S2, S3).

**Table 2:** Major biochemical components and litter quality indices of leaf litter and fine roots averaged across the four forest study sites receiving simulated nitrogen (N) deposition

Chemical characteristics	Leaf litter		Fine roots		Main effects
	Ambient	N deposition	Ambient	N deposition	
Cell‐wall fraction (%)	68.2^b^ (7.6)	62.0^a^ (8.3)	83.6^c^ (1.2)	84.4^c^ (2.2)	Type, N
AIF	15.2^b^ (1.0)	14.0^a^ (1.0)	45.1^c^ (2.0)	45.8^c^ (1.2)	Type, Site, N
Hemicellulose	14.1^ab^ (1.8)	13.8^a^ (1.3)	15.8^c^ (1.3)	15.7^bc^ (1.1)	Type
Cellulose	38.9^b^ (5.9)	34.2^b^ (6.8)	22.7^a^ (2.8)	22.9^a^ (1.9)	Type
Extractable fraction (%)	31.8^b^ (7.6)	38.0^c^ (8.3)	16.4^a^ (1.2)	15.6^a^ (2.2)	Type
Soluble phenolics	12.1^b^ (2.2)	12.5^b^ (2.1)	3.9^a^ (0.6)	3.7^a^ (0.9)	Type
Condensed tannins^†^	5.7^a^ (2.5)	4.2^a^ (1.7)	13.6^b^ (1.9)	12.4^b^ (2.8)	Type, N
NSCs	4.40^b^ (0.55)	4.94^b^ (1.11)	1.87^a^ (0.22)	1.84^a^ (0.34)	Type, Site
Lipids	7.94^b^ (1.24)	7.85^b^ (0.62)	3.60^a^ (0.45)	3.41^a^ (0.32)	Type, Site
Soluble proteins	1.11^a^ (0.20)	1.00^a^ (0.20)	3.28^b^ (0.40)	2.95^b^ (0.38)	Type, N
Unidentified^‡^	6.25^a^ (4.55)	11.75^b^ (5.79)	3.71^a^ (0.84)	3.68^a^ (1.02)	Type
N (%)	0.65^a^ (0.05)	0.81^b^ (0.18)	1.55^c^ (0.18)	1.64^c^ (0.13)	Type, Site, N
Litter quality indices (ratio)					
AIF : N	23.6^b^ (2.2)	18.1^a^ (3.58)	29.5^c^ (3.5)	28.1^c^ (2.4)	Type, Site, N
C : N	75.9^c^ (6.6)	63.3^b^ (13.3)	33.4^a^ (4.0)	31.7^a^ (3.0)	Type, Site, N
Lignocellulose index	0.22^a^ (0.02)	0.23^a^ (0.02)	0.54^b^ (0.02)	0.54^b^ (0.01)	Type

AIF, acid‐insoluble fraction; NSCs, nonstructural carbohydrates.

Values are means (SD) of three replicated plots for each treatment at each of four sites (*n *=* *12). Different letters in the same row indicate significant differences (*P* < 0.05). Significant main effects are shown (*P* < 0.05), with full statistical results in Supporting Information Table S2.

^†^Condensed tannins (CTs) are a subset of plant phenolics. There is no generally accepted CT standard for the acid‐butanol assays used to determine CTs. Thus, the CT concentrations reported here should be interpreted more as relative comparisons between fine roots and leaf litter than absolute quantification. Extractive and bound CTs were separately reported in Table S3. Bound tannins could be double‐counted in AIF in this table; however, bound CTs only represented 11.8% and 20.9% of total CTs by average in fine roots and leaf litter, respectively.

^‡^Unidentified portion is the difference between extractable fraction and the sum of soluble phenolics, NSCs, lipids and soluble proteins.

By contrast, fine roots contained a greater acid‐insoluble fraction (AIF), and more condensed tannins (CTs) and N than leaf litter (*P* < 0.001, Table 2). Fine roots averaged *c*. ×2.9 higher AIF concentrations and *c*. ×2.3 greater concentrations of CTs than those in leaf litter across four sites (Table 2). AIF was the most abundant of all eight biochemical fractions in fine root tissue, whereas cell wall polysaccharides (cellulose + hemicellulose) were the dominant constituent of leaf litter (Table 2). Fine roots had consistently higher N concentrations than leaf litter. This trend was apparent at all sites, but was strongest at site C, leading to significant interactions of site × tissue on N concentration and N‐related litter indices (*P* < 0.05, Tables S2, S3). The AIF : N ratio and lignocellulose index (LCI) were higher in fine roots than leaf litter (*P* < 0.001, Table 2).

Relative to fine roots collected in spring, autumn fine roots had lower concentrations of AIF and hemicellulose, but higher concentrations of lipids, soluble proteins and N (*P* < 0.05, Table S4). The most striking difference between spring and autumn roots was in the concentration of NSCs (Table S4): sugar increased from 10.1 ± 0.7 mg g^−1^ in spring roots to 15.7 ± 0.8 mg g^−1^ in autumn roots, whereas starch was about twice as abundant in autumn roots (data not shown).

### Biochemical fluxes

Leaf litter production across the four sites ranged from 324.0 to 447.1 g m^−2^ yr^−1^, whereas fine root litter production ranged from 175.1 to 420.1 g m^−2^ yr^−1^ (Table 3). These two types of litter production together made up 89 ± 4% of total litter production at these sites (Table 3). Simulated N deposition did not affect fine root, leaf or total litter production (*P* > 0.1, Table S5).

**Table 3:** Litter production across four northern hardwood forest study sites

Litter production (g m^−2^ yr^−1^)		Site A	Site B	Site C	Site D
Leaf litter	Ambient	324.0 (10.4)	361.9 (12.7)	399.7 (15.0)	415.2 (39.0)
	N deposition	325.7 (15.7)	376.3 (3.7)	397.9 (25.4)	447.1 (30.2)
Fine roots	Ambient	372.2 (22.6)	285.2 (30.6)	233.7 (54.9)	404.6 (124.3)
	N deposition	420.1 (10.6)	291.0 (62.7)	175.1 (22.7)	368.6 (60.1)
Total litter	Ambient	794.1 (17.0)	687.4 (43.7)	737.6 (37.1)	931.4 (114.5)
	N deposition	839.5 (27.6)	707.4 (53.1)	667.9 (50.3)	920.1 (92.8)

Values are means (SD) of three ambient plots and three N treatment plots for each site (*n *=* *3). Total litter production was the average of total aboveground litter (leaf litter, reproductive litter, and woody debris) from 1988 to 2011 for ambient plots and 1994 to 2011 for nitrogen (N) treatment plots, plus the corresponding fine root litter production. Source data are available in the Michigan Nitrogen Deposition Gradient Study database, http://webpages.uidaho.edu/nitrogen-gradient. Simulated N deposition had no effects on estimates of leaf litter, fine root or total litter production (*P *>* *0.05), whereas leaf litter, fine root and total litter production varied among sites (*P* < 0.001, Table S5).

Because the litter production of leaves and fine roots both varied among sites (Table 3), the magnitude of differences in each biochemical flux between leaf litter and fine roots also varied among sites (site × tissue; *P* < 0.01, Table S6). However, there were considerable differences between the two tissues in all biochemical fluxes except hemicellulose (Tables 4, S6). AIF and cell‐wall polysaccharides were the two largest biochemical fluxes to the soil; each of the other biochemical classes accounted for < 10% of the total litter flux. Fine roots dominated the fluxes of AIF (*c*. 71% of the total flux) and CTs (*c*. 68%) across the four sites (Table 4). Assuming that there is no meaningful N resorption during root senescence, fine roots contributed more soluble protein and N than leaves to the soil across all sites (*P* < 0.001, Table 4). By contrast, leaf litter contributed considerably more cellulose, NSCs, soluble phenolics and lipids to the soil (*P* < 0.001, Table 4).

**Table 4:** Mean flux (g m^−2^ yr^−1^) of each biochemical class to soil via leaf litter, fine roots, and their sum, followed by the proportion (%) of the combined flux of leaf litter and fine root flux contributed by fine roots

Biochemical class	Leaf litter flux		Fine root flux		Sum		Fine root (%)	
	Ambient	N	Ambient	N	Ambient	N	Ambient	N
AIF	56.7^a^ (5.6)	54.5^a^ (9.7)	146.0^b^ (44.5)	143.2^b^ (46.7)	202.8 (43.9)	197.7 (46.4)	71.0 (6.0)	70.9 (8.5)
Hemicellulose	52.7^a^ (7.9)	53.7^a^ (9.7)	51.5^a^ (17.7)	49.3^a^ (16.3)	104.2 (17.8)	102.9 (20.2)	48.5 (8.9)	46.8 (9.5)
Cellulose	145.6^b^ (23.2)	134.3^b^ (41.5)	72.5^a^ (18.1)	70.9^a^ (21.6)	218.1 (25.0)	205.2 (52.6)	33.3 (8.1)	34.8 (8.9)
Soluble phenolics	45.3^b^ (7.9)	47.7^b^ (5.0)	13.1^a^ (5.6)	12.1^a^ (5.6)	58.4 (9.9)	59.9 (4.7)	22.2 (7.0)	20.1 (8.6)
Condensed tannins^†^	20.9^a^ (7.8)	16.4^a^ (6.6)	45.0^b^ (17.0)	40.9^b^ (20.5)	65.9 (18.4)	57.3^(^ * ^)^ (16.8)	67.3 (12.0)	67.6 (18.8)
Nonstructural carbohydrates	16.5^b^ (2.6)	18.7^b^ (2.7)	6.1^a^ (2.0)	5.9^a^ (2.4)	22.6 (3.4)	24.6^(^ * ^)^ (3.0)	26.9 (6.9)	23.7 (8.2)
Lipids	29.6^b^ (4.5)	30.3^b^ (4.3)	11.9^a^ (4.4)	10.8^a^ (4.0)	41.5 (6.9)	41.1 (6.1)	28.3 (7.4)	25.8 (8.0)
Soluble proteins	4.1^a^ (0.6)	3.9^a^ (0.9)	10.5^b^ (2.5)	9.4^b^ (3.8)	14.6 (2.7)	13.3^(^ * ^)^ (3.3)	71.1 (6.6)	68.3 (13.3)
Nitrogen	2.4^a^ (0.3)	3.1^b^ (0.5)	4.9^c^ (1.0)	5.0^c^ (1.4)	7.3 (1.0)	8.1^(^ * ^)^ (1.7)	66.4 (6.1)	61.2 (7.2)

AIF, acid‐insoluble fraction.

Biochemical fluxes and proportions are shown as means (SD) of three ambient plots or three simulated nitrogen (N) deposition plots from four sites (*n *=* *12). Different letters in the same row indicate significant differences (*P* < 0.05). Marginally significant effects of N deposition on the combined flux of leaf litter and fine root flux at *P* < 0.1 (Table S7) are denoted with (*).

^†^Although *post hoc* tests did not show any significant differences of CT flux induced by simulated N deposition for either tissue type, N deposition was a significant main effect on the flux of CTs in the overall *F*‐statistics test (*P *=* *0.036, Table S6).

### Effects of simulated nitrogen deposition

Simulated N deposition generally decreased CT and increased N concentrations, as shown by significant main effects of N treatments on CT (*P *=* *0.030) and N (*P* < 0.001, Tables 2, S2). There were also significant overall effects of simulated N deposition on cell‐wall fraction, AIF, soluble proteins, AIF : N and C : N (*P* < 0.05, Table 2), but these effects were not consistent across either tissues, sites or their interactions (Tables S2, S3). The most consistent of these effects was that the decreases in C : N and AIF : N were more prominent in leaf litter than in fine roots (tissue × N: *P* < 0.05). In leaf litter at sites A, B and C, simulated N deposition decreased the average cell‐wall fraction from 69.3% to 58.1% and caused a corresponding increase in the extractive fraction (tissue × N × site: *P* < 0.02 for each; Tables S2, S3). Within the cell‐wall fraction at these sites, simulated N deposition decreased average AIF from 15.3% to 13.8% (tissue × N × site: *P *=* *0.008), but decreased cellulose from 39.9% to 30.9% (tissue × N × site: *P *=* *0.091). Within the extractive fraction at these three sites, the unidentified portion increased from an average of 5.8% to 14.3% (tissue × N × site: *P *=* *0.005). These changes did not occur in leaf litter at site D or in fine roots (Tables 2, S3).

Other effects of simulated N deposition were more idiosyncratic. Soluble protein concentrations were generally lower under simulated N deposition (*P *=* *0.018, Table 2), but this decrease did not occur at site C for leaf litter and site A for fine roots (tissue × N × site: *P *=* *0.031, Tables S2, S3). Soluble phenolics of leaf litter and fine roots showed both positive and negative responses to N deposition that depended on site (tissue × N × site: *P *=* *0.042, Tables S2, S3).

When combined with litter production, simulated N deposition increased N flux through leaf litter by an average of 29% (*P* < 0.05), but did not affect fine root N flux (*P* > 0.05, tissue × N: *P *=* *0.018, Tables 4, S6). Simulated N deposition generally decreased the fluxes of CTs (*P *=* *0.036, Tables 4, S6), but this effect was not observed for fine roots at site A and for leaf litter at site C (site × tissue × N: *P *=* *0.035, Table S6). Simulated N deposition also marginally decreased the flux of soluble protein (*P *=* *0.054), an effect that was strongest for fine roots at site C (site × tissue × N: *P *=* *0.027, Table S6). When fluxes through leaf litter and fine roots were combined, simulated N deposition marginally decreased the total fluxes of CTs and soluble proteins, and increased those of NSCs and N (*P* < 0.1, Table 4), yet this increase of N was not apparent at site C (site × N: *P *=* *0.041, Table S7). Simulated N deposition increased the cellulose flux at site D, but decreased this flux elsewhere (site × N: *P *=* *0.011, Table S7).

## Discussion

### Differences in litter biochemical composition and flux

Consistent with our hypothesis, fine roots had greater concentrations of biochemical fractions associated with chemical recalcitrance than leaf litter, and this pattern persisted across sites and N treatments. Because leaves and fine roots contributed comparable litter fluxes to the soil in these forests, the large biochemical differences meant that the flux of an individual biochemical class was often dominated by a single litter type: for example, more than two‐thirds of the fluxes of AIF and condensed tannins (CTs) were attributed to fine root turnover (Table 4). In short, leaf litter and fine roots represented fluxes of very different substrates for decomposition within these forests. Although exogenous factors also influence retention of detrital C within the soil, our observation that greater quantities of recalcitrant compounds are returned to soil through fine roots than leaf litter is consistent with previous work identifying fine roots as the major source of soil organic C (SOC).

The most striking difference between leaf litter and fine roots was that fine roots had considerably higher concentrations of acid‐insoluble fraction (AIF) than leaf litter (Table 2). The AIF has often been referred to as lignin, an aromatic heteropolymer that is difficult to degrade because its complex structure limits degradation to only nonspecific oxidative enzymes (Kirk & Farrell, 1987). Lignin has been reported to persist longer in soil than plant‐derived polysaccharides and proteins after one to several years of incubation of synthesized lignin (Martin *et al*., 1980; Stott *et al*., 1983) and plant materials (Kelleher *et al*., 2006). However, AIF isolated in this and many studies is not purely lignin, but a mixture of lignin and other complex substrates such as cutins, suberins and CT–protein complexes (Preston *et al*., 1997). AIF may still be a good indicator of chemical recalcitrance because these compounds are generally preserved in decomposing litter (Preston *et al*., 2000). The recalcitrance of AIF has been empirically demonstrated by reports that a higher initial AIF concentration or AIF : N ratio results in slower litter decomposition (Taylor *et al*., 1989; Berg, 2000; Sariyildiz & Anderson, 2003; Amin *et al*., 2014). In an empirical model derived from a 10‐yr decomposition study, AIF defined the recalcitrant litter pool, whereas the decomposition of the intermediate pool (acid‐soluble fraction) decreased when LCI increased, an effect proposed to result from the protection of cellulose by lignin (Adair *et al*., 2008). These metrics of recalcitrance (AIF concentration, AIF : N and LCI) were higher in fine roots than leaf litter, supporting the idea that fine roots are more chemically resistant to decomposition than leaf litter.

**Figure 1:**
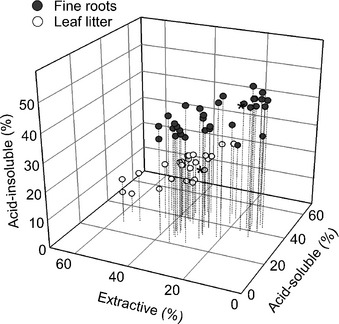
Proximate fractions of leaf litter and fine roots taken from published data across a number of boreal and temperate tree species. Extractive fraction includes relatively labile compounds consisting of both polar and nonpolar constituents. The acid‐soluble fraction approximates structural polysaccharides and the acid‐insoluble fraction includes lignin and other highly complex substrates such as cutin, suberin and complexes formed between condensed tannins and proteins. Each dot denotes the proximate fractions of a specific species/genus or functional group (e.g. a hardwood stand) in an individual study. This synthesis includes > 30 species from 14 genera. The data‐points from our study are emphasized with ‘*’. The means (SD) for three fractions in fine roots are: extractive: 24.4 (9.3), acid‐soluble: 33.6 (7.8), acid‐insoluble: 41.9 (6.9), with *n *=* *34. The means (SD) for three fractions in leaf litter are: extractive: 39.3 (9.0), acid‐soluble: 40.6 (5.5), acid‐insoluble: 18.4 (5.6), with *n *=* *26. Data references: McClaugherty *et al*. (1985); Taylor *et al*. (1989); Hendricks *et al*. (2000); Guo *et al*. (2004); Hobbie (2005); Bird & Torn (2006); Harmon *et al*. (2009); Hobbie *et al*. (2010); Solly *et al*. (2014); Sun *et al*. (2013).

In order to understand if the high concentration of AIF in fine roots was a widespread phenomenon, we compiled studies that quantified proportions of acid‐insoluble, acid‐soluble and extractive fractions across a number of boreal and temperate forests (Fig. 1), encompassing more than 30 species on three continents. These proximate fractions are frequently reported because they have been associated with increasing rates of mass loss during decomposition (acid‐insoluble < acid‐soluble < extractive; Hendricks *et al*., 2000; Adair *et al*., 2008). Fine roots had AIF concentrations that were an average of 2.3‐fold higher than in leaf litter, whereas leaf litter often had more extractive materials (Fig. 1). We conclude that chemically recalcitrant AIF is consistently more abundant in fine roots than leaf litter across a global sample of forests.

The greater CT concentrations in fine roots than leaf litter (Table 2) may add to the chemical recalcitrance of roots. Condensed tannins are less accessible to biodegradation than other plant phenolics (Bhat *et al*., 1998) and higher CT concentrations has been associated with slower decomposition (Wardle *et al*., 2002; Coq *et al*., 2010; Hättenschwiler & Jørgensen, 2010). This suppression of decay is probably because CTs can bind to proteins or cell wall components to form less degradable complexes (Cai *et al*., 1989; Northup *et al*., 1995; Mutabaruka *et al*., 2007) and because CTs can inhibit soil enzyme activity (Ushio *et al*., 2013). When the CT and AIF concentrations are combined with litter production data, it is clear that fine roots dominated the litter input of these chemically recalcitrant materials at our sites (Table 4).

Although leaf litter contained lower concentrations of CTs than fine roots, leaf litter contained higher concentrations of soluble phenolics (Table 2). Condensed tannins are a subclass of phenolics, but it is impossible to estimate the proportion of CTs in phenolics in this study because the assay we used to assess CTs provides a relative – not absolute – quantification of these compounds (see the Materials and Methods section). Aside from CTs, total phenolics also include hydrolyzable tannins (HTs) and low‐molecular‐weight phenolics, which we did not quantify separately in this study. Soil microorganisms often utilize these non‐CT phenolics as labile C sources (Schimel *et al*., 1998; Nierop *et al*., 2006) and Hättenschwiler & Jørgensen (2010) suggested that these compounds were responsible for an observed positive correlation between total phenolics and leaf litter mass loss. By contrast, Triebwasser *et al*. (2012) reported that HTs contribute significantly to soil enzyme inhibition. Leaf litter also had higher concentrations of cell‐wall polysaccharides and readily‐decomposed NSCs. The decrease of carbohydrate‐related signals in NMR spectra represented the most pronounced C loss during litter decay (Kelleher *et al*., 2006; Preston *et al*., 2009). Higher concentrations of C sources such as NSCs and polysaccharides in leaf litter than in fine roots suggest that leaf litter could be a more efficient substrate in priming decomposition.

A limitation of this study is that we used live fine roots because it was extremely difficult to identify large quantities of recently senesced fine roots. Leaf senescence is a well‐understood process that includes the breakdown of proteins, membrane lipids and nucleic acids (Lim *et al*., 2007), and which removes > 50% of foliar N and phosphorus pools (Aerts, 1996). In comparison, little is known about the senescence of fine roots because it is difficult to isolate death from decay (Comas *et al*., 2000). Resorption of nutrients during root senescence may occur, but nutrient transfers appear to be considerably smaller than those in leaves (Kunkle *et al*., 2009) and there are observations which suggest that no nutrient resorption occurs (Nambiar, 1987; Aerts, 1990; Gordon & Jackson, 2000). Notably, distal root segments have been observed to live after preceding root segments have died (Comas *et al*., 2000), making an intracellular disassembly process similar to that in leaves seem unlikely. Further, fine roots are dominated by biochemical classes that are bound in cell walls and therefore are less likely to be retranslocated during senescence.

### Implications for soil organic carbon transformation

Throughout this paper, we have used the concept of chemical recalcitrance to refer to forms of litter and biochemical fraction/classes that are resistant to mass loss in studies of plant litter decomposition. The decomposition studies that developed this concept typically track the fate of litter over months to years, or occasionally a decade (Adair *et al*., 2008). Generally, the decomposing litter in these studies has limited physical interactions with mineral soil. Within these contexts, the concept of chemical recalcitrance as a mechanism that leads to the accumulation of decomposing litter is supported both empirically (Adair *et al*., 2008; Grandy & Neff, 2008) and mechanistically (Kirk & Farrell, 1987). Our results show that in our sites and other temperate/boreal forests, fine roots are the dominant source of the recalcitrant plant biochemicals (Table 4; Fig. 1) that tend to have slow initial decay and accumulate as partially‐decomposed litter.

However, biochemical characteristics that provide the basis for the chemical recalcitrance of decomposing litter cannot *directly* explain the long‐term stabilization of C in pools associated with soil aggregates or mineral particles (Marschner *et al*., 2008). At the timescale of decades to millennia, compounds that are considered chemically recalcitrant are not preferentially preserved in soil over those considered as labile (Schmidt *et al*., 2011). Nonetheless, there is evidence for *indirect* effects of substrate biochemistry on long‐term C retention through other microbial mechanisms. Microbial products have been recognized as a major precursor to stable SOC (Mambelli *et al*., 2011; Cotrufo *et al*., 2013). More recalcitrant C fractions are generally less efficient than labile compounds in generating microbial biomass (Bahri *et al*., 2008; Blagodatskaya *et al*., 2011; Dijkstra *et al*., 2011), suggesting that labile compounds are more important for the formation of stable SOC (Cotrufo *et al*., 2013). However, fungi dominate lignin degradation (de Boer *et al*., 2005) and fungal products are thought to reside longer in soil than bacterial products (Bardgett *et al*., 2014). Understanding the manner in which substrate biochemistry affects microbial products will reveal how different biochemicals in plant debris eventually affect long‐term SOC stabilization.

### Responses to simulated nitrogen deposition

Ecosystem responses to N deposition have drawn considerable interest because human activity increased atmospheric N deposition by an order of magnitude during the last century (Galloway *et al*., 2004). At our sites, the first decade of simulated N deposition caused a 26% increase in the surface soil C pool (Pregitzer *et al*., 2008). In part, this result motivated our research in the plant biochemistry associated with initial litter decay because the partially decomposed litter in the forest floor horizon (O_e/a_) represents a distinct portion of total soil organic matter in our forests, and the slower turnover of this horizon is a major driver of soil organic matter accumulation under simulated N deposition (Zak *et al*., 2008). Other long‐term N addition experiments have also reported slower decomposition (Franklin *et al*., 2003).

Slower decomposition with increased N supply is either due to decreased initial litter quality, the inhibition of microbial activity, or both. Contrary to our second hypothesis, simulated N deposition resulted in a somewhat ‘better’ litter quality: CT concentrations generally decreased and N concentrations increased, whereas AIF concentrations and AIF : N ratios decreased in leaf litter (Table 2). At an ecosystem scale, simulated N deposition marginally decreased the total fluxes of CTs, and increased fluxes of N and NSCs entering soil (*P* < 0.1, Table 4). Thus, there is no evidence that simulated N deposition slows litter decomposition by decreasing initial litter quality. Instead, previous research at our sites observed that simulated N deposition suppressed laccase gene expression and the activity of lignin‐degrading enzymes (DeForest *et al*., 2004; Edwards *et al*., 2011).

The biochemistry of leaf litter and fine roots responded differently to simulated N deposition, supporting our third hypothesis. Simulated N deposition generally decreased the concentration of AIF and the overall cell wall fraction in leaf litter, but had little influence on any cell wall components in fine roots (Table 2). Also, simulated N deposition dramatically decreased AIF : N ratios of leaf litter, yet did not affect these ratios in fine roots. Although simulated N deposition increased leaf litter N concentration, we did not observe an increase in soluble protein (Table 2), which is dominated by Rubisco in leaves (Evans, 1989). Consistent with this, the additional foliar N induced by simulated N deposition did not increase photosynthesis at our sites (Talhelm *et al*., 2011). Additional foliar N induced by N deposition could be stored as free amino acids (Bauer *et al*., 2004), which we have not quantified. Likewise, although simulated N deposition has dramatically decreased arbuscular mycorrhizal (AM) fungal biomass and the colonization of roots by AM fungi in our sites (van Diepen *et al*., 2010), these changes were not manifest through changes in fine root biochemistry. The different response of leaf litter and fine roots to simulated N deposition indicates that the impacts of environmental change on litter biochemistry, and therefore decomposition, cannot be accurately predicted at the ecosystem‐scale solely by analyzing leaf litter.

## Supporting information

Please note: Wiley Blackwell are not responsible for the content or functionality of any supporting information supplied by the authors. Any queries (other than missing material) should be directed to the *New Phytologist* Central Office.

**Table S1** Diameter distribution for the first three orders of maple (*Acer*) roots
**Table S2** Mixed linear model analysis of biochemical traits among study sites, nitrogen (N) deposition treatments and tissue type in a split‐plot design
**Table S3** Major biochemical components and three litter quality indices of leaf litter and fine roots at each of the four forest sites receiving simulated nitrogen (N) deposition
**Table S4** Major biochemical components and three litter quality indices of spring and autumn fine roots across the four forest sites receiving simulated nitrogen (N) deposition
**Table S5** Analysis of two‐way ANOVA (site × N deposition) on the annual litter production of leaf litter, fine roots and total litter at the four northern hardwood forest study sites
**Table S6** Mixed linear model analysis of biochemical fluxes among study sites, nitrogen deposition treatments and tissue types in a split‐plot design
**Table S7** Analysis two‐way ANOVA (site × N deposition) on the combined fluxes of leaf litter and fine root biochemical fluxes at the four northern hardwood forest study sites
**Methods S1** Sequential extraction for the extractive‐free fraction.

## References

[nph13494-bib-0001] Aber JD , Melillo JM . 1982 Nitrogen immobilization in decaying hardwood leaf litter as a function of initial nitrogen and lignin content. Canadian Journal of Botany 60: 2263–2269.

[nph13494-bib-0002] Aber JD , Melillo JM , McClaugherty CA . 1990 Predicting long‐term patterns of mass loss, nitrogen dynamics, and soil organic matter formation from initial fine litter chemistry in temperate forest ecosystems. Canadian Journal of Botany 68: 2201–2208.

[nph13494-bib-0003] Abiven S , Recous S , Reyes V , Oliver R . 2005 Mineralisation of C and N from root, stem and leaf residues in soil and role of their biochemical quality. Biology and Fertility of Soils 42: 119–128.

[nph13494-bib-0004] Adair EC , Parton WJ , Del Grosso SJ , Silver WL , Harmon ME , Hall SA , Burke IC , Hart SC . 2008 Simple three‐pool model accurately describes patterns of long‐term litter decomposition in diverse climates. Global Change Biology 14: 2636–2660.

[nph13494-bib-0005] Aerts R . 1990 Nutrient use efficiency in evergreen and deciduous species from heathlands. Oecologia 84: 391–397. 10.1007/BF00329765 28313031

[nph13494-bib-0006] Aerts R . 1996 Nutrient resorption from senescing leaves of perennials: are there general patterns? Journal of Ecology 84: 597–608.

[nph13494-bib-0007] Amin BA , Chabbert ZB , Moorhead D , Bertrand I . 2014 Impact of fine litter chemistry on lignocellulolytic enzyme efficiency during decomposition of maize leaf and root in soil. Biogeochemistry 117: 169–183.

[nph13494-bib-0008] Bahri H , Rasse DP , Rumpel C , Dignac M‐F , Bardoux G , Mariotti A . 2008 Lignin degradation during a laboratory incubation followed by ^13^C isotope analysis. Soil Biology and Biochemistry 40: 1916–1922.

[nph13494-bib-0009] Bardgett RD , Mommer L , De Vries FT . 2014 Going underground: root traits as drivers of ecosystem processes. Trends in Ecology & Evolution 29: 692–699. 25459399 10.1016/j.tree.2014.10.006

[nph13494-bib-0010] Batjes NH . 1996 Total carbon and nitrogen in the soils of the world. European Journal of Soil Science 47: 151–163.

[nph13494-bib-0011] Bauer GA , Bazzaz FA , Minocha R , Long S , Magill A , Aber J , Berntson GM . 2004 Effects of chronic N additions on tissue chemistry, photosynthetic capacity, and carbon sequestration potential of a red pine (*Pinus resinosa*) stand in the NE United States. Forest Ecology and Management 196: 173–186.

[nph13494-bib-0012] Berg B . 2000 Litter decomposition and organic matter turnover in northern forest soils. Forest ecology and Management 133: 13–22.

[nph13494-bib-0013] Bhat TK , Singh B , Sharma OP . 1998 Microbial degradation of tannins‐a current perspective. Biodegradation 9: 343–357. 10192896 10.1023/a:1008397506963

[nph13494-bib-0014] Bird JA , Kleber M , Torn MS . 2008 ^13^C and ^15^N stabilization dynamics in soil organic matter fractions during needle and fine root decomposition. Organic Geochemistry 39: 465–477.

[nph13494-bib-0015] Bird JA , Torn MS . 2006 Fine roots vs. needles: a comparison of ^13^C and ^15^N dynamics in a ponderosa pine forest soil. Biogeochemistry 79: 361–382.

[nph13494-bib-0016] Blagodatskaya E , Yuyukina T , Blagodatsky S , Kuzyakov Y . 2011 Turnover of soil organic matter and of microbial biomass under C_3_–C_4_ vegetation change: consideration of ^13^C fractionation and preferential substrate utilization. Soil Biology and Biochemistry 43: 159–166.

[nph13494-bib-0017] Bligh EG , Dyer WJ . 1959 A rapid method of total lipid extraction and purification. Canadian Journal of Biochemistry and Physiology 37: 911–917. 13671378 10.1139/o59-099

[nph13494-bib-0018] de Boer W , Folman LB , Summerbell RC , Boddy L . 2005 Living in a fungal world: impact of fungi on soil bacterial niche development. FEMS Microbiology Reviews 29: 795–811. 16102603 10.1016/j.femsre.2004.11.005

[nph13494-bib-0019] Booker FL , Anttonen S , Heagle AS . 1996 Catechin, proanthocyanidin, and lignin contents of loblolly pine (*Pinus taeda*) needles after chronic exposure to ozone. New Phytologist 132: 483–492. 26763644 10.1111/j.1469-8137.1996.tb01868.x

[nph13494-bib-0020] Buchanan‐Wollaston V . 1997 The molecular biology of leaf senescence. Journal of Experimental Botany 48: 181–199.

[nph13494-bib-0021] Burton AJ , Pregitzer KS , Crawford JN , Zogg GP , Zak DR . 2004 Simulated chronic NO_3_ ^−^ deposition reduces soil respiration in northern hardwood forests. Global Change Biology 10: 1080–1091.

[nph13494-bib-0022] Burton AJ , Pregitzer KS , Hendrick RL . 2000 Relationships between fine root dynamics and nitrogen availability in Michigan northern hardwood forests. Oecologia 125: 389–399. 10.1007/s004420000455 28547334

[nph13494-bib-0023] Cai Y , Gaffney SH , Lilley TH , Haslam E . 1989 Carbohydrate–polyphenol complexation In: Hemingway RW, Karchesy JJ, eds. Chemistry and significance of condensed tannins. New York, NY, USA: Plenum Press, 307–322.

[nph13494-bib-0024] Chapin FS III , Chapin MC , Matson PA , Vitousek P . 2011 Principles of terrestrial ecosystem ecology. New York, NY, USA: Springer.

[nph13494-bib-0025] Chapman JA , King JS , Pregitzer KS , Zak DR . 2005 Effects of elevated concentrations of atmospheric CO_2_ and tropospheric O_3_ on decomposition of fine roots. Tree Physiology 25: 1501–1510. 16137936 10.1093/treephys/25.12.1501

[nph13494-bib-0026] Chávez AL , Bedoya J , Sánchez T , Iglesias C , Ceballos H , Roca W . 2000 Iron, carotene, and ascorbic acid in cassava roots and leaves. Food & Nutrition Bulletin 21: 410–413.

[nph13494-bib-0027] Chen Z , Gallie DR . 2005 Increasing tolerance to ozone by elevating foliar ascorbic acid confers greater protection against ozone than increasing avoidance. Plant Physiology 138: 1673–1689. 15951482 10.1104/pp.105.062000 PMC1176437

[nph13494-bib-0028] Chow PS , Landhäusser SM . 2004 A method for routine measurements of total sugar and starch content in woody plant tissues. Tree Physiology 24: 1129–1136. 15294759 10.1093/treephys/24.10.1129

[nph13494-bib-0029] Comas LH , Eissenstat DM , Lakso AN . 2000 Assessing root death and root system dynamics in a study of grape canopy pruning. New Phytologist 147: 171–178.

[nph13494-bib-0030] Coq S , Souquet JM , Meudec E , Cheynier V , Hättenschwiler S . 2010 Interspecific variation in leaf litter tannins drives decomposition in a tropical rain forest of French Guiana. Ecology 91: 2080–2091. 20715630 10.1890/09-1076.1

[nph13494-bib-0031] Cornwell WK , Cornelissen JH , Amatangelo K , Dorrepaal E , Eviner VT , Godoy O , Hobbie SE , Hoorens B , Kurokawa H , Parez‐Harguindeguy N *et al* 2008 Plant species traits are the predominant control on litter decomposition rates within biomes worldwide. Ecology Letters 11: 1065–1071. 18627410 10.1111/j.1461-0248.2008.01219.x

[nph13494-bib-0032] Cotrufo MF , Wallenstein MD , Boot CM , Denef K , Paul E . 2013 The Microbial Efficiency‐Matrix Stabilization (MEMS) framework integrates plant litter decomposition with soil organic matter stabilization: do labile plant inputs form stable soil organic matter? Global Change Biology 19: 988–995. 23504877 10.1111/gcb.12113

[nph13494-bib-0033] Cyr D , Buxton G , Webb D , Dumbroff E . 1990 Accumulation of free amino acids in the shoots and roots of three northern conifers during drought. Tree Physiology 6: 293–303. 14972940 10.1093/treephys/6.3.293

[nph13494-bib-0034] Dearden FM , Dehlin H , Wardle DA , Nilsson MC . 2006 Changes in the ratio of twig to foliage in litterfall with species composition, and consequences for decomposition across a long term chronosequence. Oikos 115: 453–462.

[nph13494-bib-0035] DeForest JL , Zak DR , Pregitzer KS , Burton AJ . 2004 Nitrate deposition and the microbial degradation of cellulose and lignin in a northern hardwood forest. Soil Biology & Biochemistry 36: 965–971.

[nph13494-bib-0036] Dickson RE . 1979 Analytical procedures for the sequential extraction of ^14^C‐labeled constituents from leaves, bark and wood of cottonwood plants. Physiologia Plantarum 45: 480–488.

[nph13494-bib-0037] van Diepen LT , Lilleskov EA , Pregitzer KS , Miller RM . 2010 Simulated nitrogen deposition causes a decline of intra‐and extraradical abundance of arbuscular mycorrhizal fungi and changes in microbial community structure in northern hardwood forests. Ecosystems 13: 683–695.

[nph13494-bib-0038] Dijkstra P , Thomas SC , Heinrich PL , Koch GW , Schwartz E , Hungate BA . 2011 Effect of temperature on metabolic activity of intact microbial communities: evidence for altered metabolic pathway activity but not for increased maintenance respiration and reduced carbon use efficiency. Soil Biology and Biochemistry 43: 2023–2031.

[nph13494-bib-0039] Edwards IP , Zak DR , Kellner H , Eisenlord SD , Pregitzer KS . 2011 Simulated atmospheric N deposition alters fungal community composition and suppresses lignocellulolytic gene expression in forest floor of a northern hardwood forest. PLoS ONE 6: e20421. 21701691 10.1371/journal.pone.0020421 PMC3119081

[nph13494-bib-0040] Evans JR . 1989 Photosynthesis and nitrogen relationships in leaves of C_3_ plants. Oecologia 78: 9–19. 10.1007/BF00377192 28311896

[nph13494-bib-0041] Franklin O , Högberg P , Ekblad A , Ågren GI . 2003 Pine forest floor carbon accumulation in response to N and PK additions: bomb ^14^C modelling and respiration studies. Ecosystems 6: 644–658.

[nph13494-bib-0042] Freschet GT , Cornwell WK , Wardle DA , Elumeeva TG , Liu W , Jackson BG , Onipchenko VG , Soudzilovskaia NA , Tao J , Cornelissen JHC *et al* 2013 Linking litter decomposition of above‐ and below‐ground organs to plant–soil feedbacks worldwide. Journal of Ecology 101: 943–952.

[nph13494-bib-0043] Galloway JN , Dentener FJ , Capone DG , Boyer EW , Howarth RW , Seitzinger SP , Asner GP , Cleveland CC , Green PA , Holland EA *et al* 2004 Nitrogen cycles: past, present and future. Biogeochemistry 70: 153–226.

[nph13494-bib-0044] Gergoff G , Chaves A , Bartoli CG . 2010 Ethylene regulates ascorbic acid content during dark‐induced leaf senescence. Plant Science 178: 207–212.

[nph13494-bib-0045] Gholz HL , Wedin DA , Smitherman SM , Harmon ME , Parton WJ . 2000 Long‐term dynamics of pine and hardwood litter in contrasting environments: toward a global model of decomposition. Global Change Biology 6: 751–765.

[nph13494-bib-0046] Gill RA , Jackson RB . 2000 Global patterns of root turnover for terrestrial ecosystems. New Phytologist 147: 13–31.

[nph13494-bib-0047] Gordon WS , Jackson RB . 2000 Nutrient concentrations in fine roots. Ecology 81: 275–280.

[nph13494-bib-0048] Grandy AS , Neff JC . 2008 Molecular C dynamics downstream: the biochemical decomposition sequence and its impact on soil organic matter structure and function. Science of the Total Environment 404: 297–307. 18190951 10.1016/j.scitotenv.2007.11.013

[nph13494-bib-0049] Grier CC , Vogt KA , Keyes MR , Edmonds RL . 1981 Biomass distribution and above‐and below‐ground production in young and mature *Abies amabilis* zone ecosystems of the Washington Cascades. Canadian Journal of Forest Research 11: 155–167.

[nph13494-bib-0050] Guo DL , Mitchell RJ , Hendricks JJ . 2004 Fine root branch orders respond differentially to carbon source‐sink manipulations in a longleaf pine forest. Oecologia 140: 450–457. 15179577 10.1007/s00442-004-1596-1

[nph13494-bib-0051] Guo DL , Xia MX , Wei X , Chang WJ , Liu Y , Wang ZQ . 2008 Anatomical traits associated with absorption and mycorrhizal colonization are linked to root branch order in twenty‐three Chinese temperate tree species. New Phytologist 180: 673–683. 18657210 10.1111/j.1469-8137.2008.02573.x

[nph13494-bib-0052] Harmon ME , Silver WL , Fasth B , Chen H , Burke IC , Parton WJ , Hart SC , Currie WS . 2009 Long‐term patterns of mass loss during the decomposition of leaf and fine root litter: an intersite comparison. Global Change Biology 15: 1320–1338.

[nph13494-bib-0053] Hättenschwiler S , Coq S , Barantal S , Handa IT . 2011 Leaf traits and decomposition in tropical rainforests: revisiting some commonly held views and towards a new hypothesis. New Phytologist 189: 950–965. 21374832 10.1111/j.1469-8137.2010.03483.x

[nph13494-bib-0054] Hättenschwiler S , Jørgensen HB . 2010 Carbon quality rather than stoichiometry controls litter decomposition in a tropical rain forest. Journal of Ecology 98: 754–763.

[nph13494-bib-0055] Hendricks JJ , Aber JD , Nadelhoffer KJ , Hallett RD . 2000 Nitrogen controls on fine root substrate quality in temperate forest ecosystems. Ecosystems 3: 57–69.

[nph13494-bib-0056] Hobbie SE . 2005 Contrasting effects of substrate and fertilizer nitrogen on the early stages of litter decomposition. Ecosystems 8: 644–656.

[nph13494-bib-0057] Hobbie SE , Chapin FS III . 1998 The response of tundra plant biomass, aboveground production, nitrogen, and CO_2_ flux to experimental warming. Ecology 79: 1526–1544.

[nph13494-bib-0058] Hobbie SE , Oleksyn J , Eissenstat DM , Reich PB . 2010 Fine root decomposition rates do not mirror those of leaf litter among temperate tree species. Oecologia 162: 505–513. 19882174 10.1007/s00442-009-1479-6

[nph13494-bib-0059] Holland EA , Braswell BH , Sulzman J , Lamarque JF . 2005 Nitrogen deposition onto the United States and Western Europe: synthesis of observations and models. Ecological Applications 15: 38–57.

[nph13494-bib-0060] van Huysen TL , Harmon ME , Perakis SS , Chen H . 2013 Decomposition and nitrogen dynamics of ^15^N‐labeled leaf, root, and twig litter in temperate coniferous forests. Oecologia 173: 1563–1573. 23884664 10.1007/s00442-013-2706-8

[nph13494-bib-0061] Iiyama K , Lam TBT , Stone BA . 1994 Covalent cross‐links in the cell wall. Plant Physiology 104: 315–320. 12232082 10.1104/pp.104.2.315 PMC159201

[nph13494-bib-0062] Jones CG , Hare JD , Compton SJ . 1989 Measuring plant protein with the Bradford assay. Journal of chemical ecology 15: 979–992. 24271900 10.1007/BF01015193

[nph13494-bib-0063] Joslin J , Gaudinski JB , Torn MS , Riley W , Hanson PJ . 2006 Fine‐root turnover patterns and their relationship to root diameter and soil depth in a 14C‐labeled hardwood forest. New Phytologist 172: 523–535. 17083682 10.1111/j.1469-8137.2006.01847.x

[nph13494-bib-0064] Kelleher BP , Simpson MJ , Simpson AJ . 2006 Assessing the fate and transformation of plant residues in the terrestrial environment using HR‐MAS NMR spectroscopy. Geochimica et Cosmochimica Acta 70: 4080–4094.

[nph13494-bib-0065] Kirk TK , Farrell RL . 1987 Enzymatic “combustion”: the microbial degradation of lignin. Annual Reviews in Microbiology 41: 465–501. 10.1146/annurev.mi.41.100187.002341 3318677

[nph13494-bib-0066] Kramer C , Trumbore S , Fröberg M , Cisneros Dozal LM , Zhang D , Xu X , Santos GM , Hanson PJ . 2010 Recent (<4 year old) leaf litter is not a major source of microbial carbon in a temperate forest mineral soil. Soil Biology and Biochemistry 42: 1028–1037.

[nph13494-bib-0067] Kunkle JM , Walters MB , Kobe RK . 2009 Senescence‐related changes in nitrogen in fine roots: mass loss affects estimation. Tree Physiology 29: 715–723. 19203982 10.1093/treephys/tpp004

[nph13494-bib-0068] Lehmann J , Schroth G , Zech W . 1995 Decomposition and nutrient release from leaves, twigs and roots of three alley‐cropped tree legumes in central Togo. Agroforestry Systems 29: 21–36.

[nph13494-bib-0069] Li A , Guo D , Wang Z , Liu H . 2010a Nitrogen and phosphorus allocation in leaves, twigs, and fine roots across 49 temperate, subtropical and tropical tree species: a hierarchical pattern. Functional Ecology 24: 224–232.

[nph13494-bib-0070] Li C , Trombley JD , Schmidt MA , Hagerman AE . 2010b Preparation of an acid butanol standard from fresh apples. Journal of Chemical Ecology 36: 453–460. 20379766 10.1007/s10886-010-9784-4

[nph13494-bib-0071] Lim PO , Kim HJ , Gil Nam H . 2007 Leaf senescence. Annual Review of Plant Biology 58: 115–136. 10.1146/annurev.arplant.57.032905.105316 17177638

[nph13494-bib-0072] Littell RC , Milliken GA , Stroup WW , Wolfinger RD , Schabenberger O . 2006 SAS for mixed models. Cary, NC, USA: SAS Institute Inc.

[nph13494-bib-0073] Loya WM , Johnson LC , Nadelhoffer KJ . 2004 Seasonal dynamics of leaf‐and root‐derived C in arctic tundra mesocosms. Soil Biology and Biochemistry 36: 655–666.

[nph13494-bib-0700] MacDonald NW , Burton AJ , Liechty HO , Witter JO , Pregitzer KS , Mroz GD , Richter DD . 1992 Ion leaching in forest ecosystems along a Great Lakes air pollution gradient. Journal of Environmental Quality 21: 614–623.

[nph13494-bib-0074] Mambelli S , Bird JA , Gleixner G , Dawson TE , Torn MS . 2011 Relative contribution of foliar and fine root pine litter to the molecular composition of soil organic matter after *in situ* degradation. Organic Geochemistry 42: 1099–1108.

[nph13494-bib-0075] Marschner B , Brodowski S , Dreves A , Gleixner G , Gude A , Grootes PM , Hamer U , Heim A , Jandl J , Ji R *et al* 2008 How relevant is recalcitrance for the stabilization of organic matter in soils? Journal of Plant Nutrition and Soil Science 171: 91–110.

[nph13494-bib-0076] Martin JP , Haider K , Kassim G . 1980 Biodegradation and stabilization after 2 years of specific crop, lignin, and polysaccharide carbons in soils. Soil Science Society of America Journal 44: 1250–1255.

[nph13494-bib-0077] McClaugherty CA , Pastor J , Aber JD , Melillo JM . 1985 Forest litter decomposition in relation to soil nitrogen dynamics and litter quality. Ecology 66: 266–275.

[nph13494-bib-0078] McCormack ML , Dicke IA , Eissenstat DM , Fahey TJ , Fernandez CW , Guo D , Helmisaari H‐S , Hobbie EA , Iversen CM , Jackson RB *et al.* 2015 Redefining fine roots improves understanding of below‐ground contributions to terrestrial biosphere processes. New Phytologist 207: 505–518. 25756288 10.1111/nph.13363

[nph13494-bib-0079] McGuire A , Sitch S , Clein J , Dargaville R , Esser G , Foley J , Heimann M , Joos F , Kaplan J , Kicklighter DW *et al* 2001 Carbon balance of the terrestrial biosphere in the twentieth century: analyses of CO_2_, climate and land use effects with four process‐based ecosystem models. Global Biogeochemical Cycles 15: 183–206.

[nph13494-bib-0080] Melillo JM , Aber JD , Muratore JF . 1982 Nitrogen and lignin control of hardwood leaf litter decomposition dynamics. Ecology 63: 621–626.

[nph13494-bib-0081] Mutabaruka R , Hairiah K , Cadisch G . 2007 Microbial degradation of hydrolysable and condensed tannin polyphenol–protein complexes in soils from different land‐use histories. Soil Biology and Biochemistry 39: 1479–1492.

[nph13494-bib-0082] Nambiar EKS . 1987 Do nutrients translocate from fine roots? Canadian Journal of Forest Research 17: 913–918.

[nph13494-bib-0083] Nierop KG , Verstraten JM , Tietema A , Westerveld JW , Wartenbergh PE . 2006 Short‐and long‐term tannin induced carbon, nitrogen and phosphorus dynamics in Corsican pine litter. Biogeochemistry 79: 275–296.

[nph13494-bib-0084] Northup RR , Yu Z , Dahlgren RA , Vogt KA . 1995 Polyphenol control of nitrogen release from pine litter. Nature 377: 227–229.

[nph13494-bib-0085] Ostertag R , Hobbie SE . 1999 Early stages of root and leaf decomposition in Hawaiian forests: effects of nutrient availability. Oecologia 121: 564–573. 10.1007/s004420050963 28308366

[nph13494-bib-0086] Paul EA . 2006 Soil microbiology, ecology and biochemistry. San Diego, CA, USA: Academic Press.

[nph13494-bib-0087] Pregitzer KS , Burton AJ , Zak DR , Talhelm AF . 2008 Simulated chronic nitrogen deposition increases carbon storage in Northern Temperate forests. Global Change Biology 14: 142–153.

[nph13494-bib-0088] Pregitzer KS , DeForest JL , Burton AJ , Allen MF , Ruess RW , Hendrick RL . 2002 Fine root architecture of nine North American trees. Ecological Monographs 72: 293–309.

[nph13494-bib-0089] Preston CM , Nault JR , Trofymow J . 2009 Chemical changes during 6 years of decomposition of 11 litters in some Canadian forest sites. Part 2. ^13^C abundance, solid‐state ^13^C NMR spectroscopy and the meaning of “lignin”. Ecosystems 12: 1078–1102.

[nph13494-bib-0090] Preston CM , Trofymow J , the Canadian Intersite Decomposition Experiment Working Group . 2000 Variability in litter quality and its relationship to litter decay in Canadian forests. Canadian Journal of Botany 78: 1269–1287.

[nph13494-bib-0091] Preston CM , Trofymow J , Niu J , Sayer BG . 1997 ^13^C nuclear magnetic resonance spectroscopy with cross‐polarization and magic‐angle spinning investigation of the proximate‐analysis fractions used to assess litter quality in decomposition studies. Canadian Journal of Botany 75: 1601–1613.

[nph13494-bib-0092] Raich J , Schlesinger WH . 1992 The global carbon dioxide flux in soil respiration and its relationship to vegetation and climate. Tellus Series B 44: 81–99.

[nph13494-bib-0093] Rasse DP , Rumpel C , Dignac M‐F . 2005 Is soil carbon mostly root carbon? Mechanisms for a specific stabilisation. Plant and Soil 269: 341–356.

[nph13494-bib-0094] Sariyildiz T , Anderson JM . 2003 Interactions between litter quality, decomposition and soil fertility: a laboratory study. Soil Biology and Biochemistry 35: 391–399.

[nph13494-bib-0095] Schimel JP , Cates RG , Ruess R . 1998 The role of balsam poplar secondary chemicals in controlling soil nutrient dynamics through succession in the Alaskan taiga. Biogeochemistry 42: 221–234.

[nph13494-bib-0096] Schmidt MW , Torn MS , Abiven S , Dittmar T , Guggenberger G , Janssens IA , Kleber M , Kogel‐Knabner I , Lehmann J , Manning DA *et al* 2011 Persistence of soil organic matter as an ecosystem property. Nature 478: 49–56. 21979045 10.1038/nature10386

[nph13494-bib-0097] Singleton VL , Orthofer R , Lamuela‐Raventos RM . 1999 Analysis of total phenols and other oxidation substrates and antioxidants by means of Folin‐Ciocalteu reagent. Methods in Enzymology 299: 152–178.

[nph13494-bib-0098] Six J , Conant RT , Paul EA , Paustian K . 2002 Stabilization mechanisms of soil organic matter: implications for C‐saturation of soils. Plant and Soil 241: 155–176.

[nph13494-bib-0099] Smedes F , Thomasen TK . 1996 Evaluation of the Bligh & Dyer lipid determination method. Marine Pollution Bulletin 32: 681–688.

[nph13494-bib-0100] Solly EF , Schöning I , Boch S , Kandeler E , Marhan S , Michalzik B , Schrumpf M . 2014 Factors controlling decomposition rates of fine root litter in temperate forests and grasslands. Plant and Soil 382: 203–218.

[nph13494-bib-0101] Stott DE , Kassim G , Jarrell W , Martin J , Haider K . 1983 Stabilization and incorporation into biomass of specific plant carbons during biodegradation in soil. Plant and Soil 70: 15–26.

[nph13494-bib-0102] Sun T , Mao Z , Han Y . 2013 Slow decomposition of very fine roots and some factors controlling the process: a 4‐year experiment in four temperate tree species. Plant and Soil 372: 445–458.

[nph13494-bib-0103] Talhelm AF , Burton AJ , Pregitzer KS , Campione MA . 2013 Chronic nitrogen deposition reduces the abundance of dominant forest understory and groundcover species. Forest Ecology and Management 293: 39–48.

[nph13494-bib-0104] Talhelm AF , Pregitzer KS , Burton AJ . 2011 No evidence that chronic nitrogen additions increase photosynthesis in mature sugar maple forests. Ecological Applications 21: 2413–2424. 22073632 10.1890/10-2076.1

[nph13494-bib-0105] Talhelm AF , Pregitzer KS , Burton AJ , Zak DR . 2012 Air pollution and the changing biogeochemistry of northern forests. Frontiers in Ecology and the Environment 10: 181–185.

[nph13494-bib-0106] Taylor BR , Parkinson D , Parsons WF . 1989 Nitrogen and lignin content as predictors of litter decay rates: a microcosm test. Ecology 70: 97–104.

[nph13494-bib-0107] Taylor BR , Prescott CE , Parsons W , Parkinson D . 1991 Substrate control of litter decomposition in four Rocky Mountain coniferous forests. Canadian Journal of Botany 69: 2242–2250.

[nph13494-bib-0108] Triebwasser DJ , Tharayil N , Preston CM , Gerard PD . 2012 The susceptibility of soil enzymes to inhibition by leaf litter tannins is dependent on the tannin chemistry, enzyme class and vegetation history. New Phytologist 196: 1122–1132. 23025512 10.1111/j.1469-8137.2012.04346.x

[nph13494-bib-0109] Tschaplinski T , Stewart D , Norby R . 1995 Interactions between drought and elevated CO_2_ on osmotic adjustment and solute concentrations of tree seedlings. New Phytologist 131: 169–177. 10.1111/j.1469-8137.1995.tb03010.x 33874415

[nph13494-bib-0110] Ushio M , Balser TC , Kitayama K . 2013 Effects of condensed tannins in conifer leaves on the composition and activity of the soil microbial community in a tropical montane forest. Plant and Soil 365: 157–170.

[nph13494-bib-0111] Valenzuela‐Estrada LR , Vera‐Caraballo V , Ruth LE , Eissenstat DM . 2008 Root anatomy, morphology, and longevity among root orders in *Vaccinium corymbosum* (Ericaceae*)* . American Journal of Botany 95: 1506–1514. 21628158 10.3732/ajb.0800092

[nph13494-bib-0112] Vivanco L , Austin AT . 2006 Intrinsic effects of species on leaf litter and root decomposition: a comparison of temperate grasses from North and South America. Oecologia 150: 97–107. 16917779 10.1007/s00442-006-0495-z

[nph13494-bib-0113] Waid JS . 1974 Decomposition of roots In: Dickinson CH, Pugh GJF, eds. Biology of plant litter decomposition, *vol* 1 London, UK: Academic Press, 175–211.

[nph13494-bib-0114] Waldrop MP , Firestone MK . 2004 Microbial community utilization of recalcitrant and simple carbon compounds: impact of oak‐woodland plant communities. Oecologia 138: 275–284. 14614618 10.1007/s00442-003-1419-9

[nph13494-bib-0115] Wardle DA , Bonner KI , Barker GM . 2002 Linkages between plant litter decomposition, litter quality, and vegetation responses to herbivores. Functional Ecology 16: 585–595.

[nph13494-bib-0116] Wickings K , Grandy AS , Reed SC , Cleveland CC . 2012 The origin of litter chemical complexity during decomposition. Ecology Letters 15: 1180–1188. 22897741 10.1111/j.1461-0248.2012.01837.x

[nph13494-bib-0117] Xia MX , Guo DL , Pregitzer KS . 2010 Ephemeral root modules in *Fraxinus mandshurica* . New Phytologist 188: 1065–1074. 21058949 10.1111/j.1469-8137.2010.03423.x

[nph13494-bib-0118] Yu Z , Dahlgren RA . 2000 Evaluation of methods for measuring polyphenols in conifer foliage. Journal of Chemical Ecology 26: 2119–2140.

[nph13494-bib-0119] Zak DR , Holmes WE , Burton AJ , Pregitzer KS , Talhelm AF . 2008 Simulated atmospheric NO_3_ ^‐^ deposition increases soil organic matter by slowing decomposition. Ecological applications 18: 2016–2027. 19263894 10.1890/07-1743.1

[nph13494-bib-0120] Zhang D , Hui D , Luo Y , Zhou G . 2008 Rates of litter decomposition in terrestrial ecosystems: global patterns and controlling factors. Journal of Plant Ecology 1: 85–93.

