# Supplementary material for: Fine roots are the dominant source of recalcitrant plant litter in sugar maple‐dominated northern hardwood forests
Source: New Phytol. 2015 Jun 12;208(3):715–26. doi: 10.1111/nph.13494 (PMC5033015; doi:10.1111/nph.13494)
Supplement: Supplementary file 1 — Table S1 Diameter distribution for the first three orders of maple (Acer) roots Table S2 Mixed linear model analysis of biochemical traits among study sites, nitrogen (N) deposition treatments and tissue type in a split‐plot design Table S3 Major biochemical components and three litter quality indices of leaf litter and fine roots at each of the four forest sites receiving simulated nitrogen (N) deposition Table S4 Major biochemical components and three litter quality indices of spring and autumn fine roots across the four forest sites receiving simulated nitrogen (N) deposition Table S5 Analysis of two‐way ANOVA (site × N deposition) on the annual litter production of leaf litter, fine roots and total litter at the four northern hardwood forest study sites Table S6 Mixed linear model analysis of biochemical fluxes among study sites, nitrogen deposition treatments and tissue types in a split‐plot design Table S7 Analysis two‐way ANOVA (site × N deposition) on the combined fluxes of leaf litter and fine root biochemical fluxes at the four northern hardwood forest study sites Methods S1 Sequential extraction for the extractive‐free fraction. [file NPH-208-715-s001.pdf]

New Phytologist **Supporting Information**

Fine roots are the dominant source of recalcitrant plant litter in sugar maple dominated northern hardwood forests

Mengxue Xia, Alan F. Talhelm and Kurt S. Pregitzer

Article acceptance date: 3 May 2015

The following Supporting Information is available for this article:

Table S1 Diameter (mm) distribution for the first three orders of maple (*Acer*) roots

Table S2 Mixed linear model analysis of biochemical traits among study sites, nitrogen (N) deposition treatments, and tissue type in a split-plot design

Table S3 Major biochemical components and three litter quality indices of leaf litter and fine roots at each of the four forest sites receiving simulated nitrogen (N) deposition

Table S4 Major biochemical components and three litter quality indices of spring and autumn fine roots across the four forest sites receiving simulated nitrogen (N) deposition

Table S5 Analysis of two-way ANOVA (site × N deposition) on the annual litter production of leaf litter, fine roots, and total litter at the four northern hardwood forest study sites

Table S6 Mixed linear model analysis of biochemical fluxes among study sites, nitrogen deposition treatments, and tissue types in a split-plot design

Table S7 Analysis two-way ANOVA (site × N deposition) on the combined fluxes of leaf litter and fine root biochemical fluxes at the four northern hardwood forest study sites

Methods S1 Sequential extraction for the extractive-free fraction

Table S1 Diameter (mm) distribution for the first three orders of maple (*Acer*) roots

Root order	Root diameter	
	Average	*n*
Order 1	0.26 (0.08)	1517
Order 2	0.26 (0.05)	1507
Order 3	0.30 (0.11)	383

The average diameter (SD) of each order were derived from root segments in three ambient plots in each of the four study sites. For each plot, approximately five intact large root branches were randomly selected when roots were excavated according to the procedure described from the Materials and Methods section. For each large root branch, five to six root segments of Order 3 and *c*. 20 segments of Order 1 and 2 were carefully dissected from their mother branches according to the procedures of Pregitzer *et al.* (2002) and measured for diameter with a dissecting microscope (Zeiss, Oberkochen, Germany).

Reference

Pregitzer KS, DeForest JL, Burton AJ, Allen MF, Ruess RW, Hendrick RL. 2002. Fine root architecture of nine North American trees. *Ecological Monographs* **72**: 293–309.

Table S2 Mixed linear model analysis of biochemical traits among study sites, nitrogen (N) deposition treatments, and tissue type in a split-plot design

Chemical characteristics	Whole-plot						Within-plot							
	Site df = 3 / 16		N deposition df = 1 / 16		Site × N df = 3 / 16		Tissue df =1 / 16		Tissue × site df =3 / 16		Tissue × N df =1 / 16		Tissue × site × N df =3 / 16	
	F	*P*	*F*	*P*	*F*	*P*	*F*	*P*	*F*	*P*	*F*	*P*	*F*	*P*
Cell-wall fraction	2.54	0.093	4.88	**0.042**	4.13	**0.024**	188.35	**<0.001**	2.76	0.076	7.61	**0.014**	4.82	**0.014**
AIF	4.28	**0.021**	6.55	**0.021**	0.83	0.496	8435	**<0.001**	3.25	0.050	14.61	**0.002**	5.65	**0.008**
Hemicellulose	0.38	0.771	0.11	0.745	0.16	0.924	18.85	**<0.001**	1.29	0.311	0.03	0.874	0.59	0.630
Cellulose	1.00	0.418	2.75	0.117	5.07	**0.012**	216.16	**<0.001**	3.45	**0.042**	5.73	**0.029**	2.56	0.091
Extractive fraction	1.66	0.214	1.94	0.183	3.70	**0.034**	314.92	**<0.001**	3.22	0.051	7.62	**0.014**	4.53	**0.018**
Soluble phenolics	3.15	0.054	0.21	0.655	0.92	0.456	940.61	**<0.001**	10.60	**<0.001**	1.70	0.210	3.45	**0.042**
Condensed tannins	1.36	0.289	5.71	**0.030**	0.78	0.520	161.38	**<0.001**	2.88	0.068	1.14	0.301	2.78	0.075
NSCs	5.51	**0.009**	0.90	0.358	1.04	0.403	514.55	**<0.001**	2.38	0.108	2.37	0.143	1.15	0.361
Lipids	5.48	**0.009**	0.74	0.403	1.36	0.290	670.10	**<0.001**	0.42	0.740	0.62	0.443	0.49	0.695
Soluble proteins	1.40	0.278	6.93	**0.018**	0.13	0.942	730.71	**<0.001**	0.61	0.621	0.32	0.582	3.81	**0.031**
Unidentified	1.14	0.362	3.61	0.076	4.13	**0.024**	30.26	**<0.001**	4.13	**0.024**	6.04	**0.026**	6.35	**0.005**
N	7.63	**0.002**	20.43	**<0.001**	2.04	0.149	946.14	**<0.001**	7.82	**0.002**	4.06	0.061	1.41	0.277
AIF : N	4.16	**0.023**	26.65	**<0.001**	2.54	0.093	114.83	**<0.001**	3.29	**0.048**	14.04	**0.002**	2.55	0.093
C : N	10.11	**<0.001**	19.63	**<0.001**	2.14	0.135	790.56	**<0.001**	7.53	**0.002**	7.99	**0.012**	2.15	0.134
Lignocellulose index	0.36	0.783	0.49	0.492	3.37	**0.045**	3643	**<0.001**	1.72	0.204	0.02	0.901	2.18	0.130

The mixed linear models included fixed effects shown in the table and random effects of plots nested (n = 3) in (site × N treatment). The degrees of freedom are shown as ‘numerator df / denominator df’. Bold numbers: $P < 0.05$. AIF, acid-insoluble fraction; NSCs, non-structural carbohydrates.

Table S3 Major biochemical components and three litter quality indices of leaf litter and fine roots at each of the four forest sites receiving simulated nitrogen (N) deposition

Chemical characteristics	Site A				Site B			
	Leaf litter		Fine roots		Leaf litter		Fine roots	
	Ambient	N deposition	Ambient	N deposition	Ambient	N deposition	Ambient	N deposition
Cell-wall fraction (%)	70.64 (14.33)	55.59 (1.92)*	82.49 (1.18)	81.91 (0.51)	69.99 (5.77)	64.07 (3.33)	84.32 (0.50)	86.04 (0.22)
AIF	15.54 (0.99)	12.80 (0.24)**	42.71 (1.09)	45.49 (1.76)	15.84 (1.28)	14.91 (0.47)	45.34 (0.49)	45.72 (0.81)
Hemicellulose	15.09 (3.64)	13.91 (0.56)	14.78 (0.99)	15.43 (0.54)	13.60 (1.24)	13.43 (1.39)	16.70 (1.24)	16.02 (1.02)
Cellulose	40.01 (11.26)	28.88 (1.67)	25.01 (0.18)	21.00 (1.11)	40.55 (4.47)	35.73 (2.14)	22.28 (1.99)	24.30 (1.73)
Extractable fraction (%)	29.36 (14.33)	44.41 (1.92)*	17.51 (1.18)	18.09 (0.51)	30.01 (5.77)	35.93 (3.33)	15.68 (0.50)	13.96 (0.22)
Soluble phenolics	12.86 (2.54)	14.55 (0.89)	3.88 (0.21)	4.03 (0.11)	13.26 (2.52)	12.43 (1.25)	3.24 (0.24)	2.99 (0.34)
Condensed tannins	7.53 (2.58)	3.48 (0.73)	14.83 (0.77)	15.82 (1.13)	6.05 (4.17)	4.92 (2.55)	11.70 (1.25)	10.49 (0.50)
Extractable fraction	6.80 (2.55)	3.17 (0.70)	11.75 (0.61)	12.89 (1.27)	5.51 (4.06)	4.46 (2.47)	9.11 (1.18)	7.97 (0.67)
Bound fraction	0.73 (0.15)	0.31 (0.07)*	3.08 (0.16)	2.93 (0.17)	0.53 (0.11)	0.46 (0.10)	2.59 (0.19)	2.52 (0.20)
NSCs	4.81 (0.67)	6.04 (0.59)	2.09 (0.08)	2.10 (0.32)	4.31 (0.24)	4.46 (0.17)	1.73 (0.24)	1.71 (0.10)
Lipids	8.98 (1.50)	8.21 (0.27)	3.72 (0.29)	3.54 (0.33)	7.47 (1.33)	7.41 (0.50)	3.72 (0.21)	3.18 (0.43)
Soluble proteins	1.28 (0.22)	0.91 (0.18)	3.20 (0.07)	3.30 (0.26)	1.06 (0.30)	1.02 (0.16)	3.24 (0.25)	2.61 (0.28)
Unidentified	4.18 (7.24)	14.70 (0.56)*	4.61 (0.94)	5.13 (0.37)	3.90 (1.75)	10.62 (1.80)	3.75 (0.47)	3.47 (0.84)
N (%)	0.63 (0.04)	0.92 (0.27)*	1.35 (0.02)	1.55 (0.06)	0.70 (0.02)	0.94 (0.07)	1.74 (0.11)	1.72 (0.06)
Litter quality indices (ratio)								
AIF : N	24.61 (1.87)	14.73 (4.18)**	31.73 (0.68)	29.42 (1.44)	22.47 (1.21)	15.96 (0.78)(*)	26.30 (1.92)	26.63 (1.31)
C : N	78.22 (3.66)	55.38 (15.01)*	37.38 (0.59)	33.66 (1.43)	67.56 (1.94)	50.81 (4.07) (*)	29.05 (1.80)	29.32 (1.02)
Lignocellulose index	0.23 (0.04)	0.23 (0.004)	0.52 (0.01)	0.56 (0.02)	0.23 (0.02)	0.23 (0.02)	0.54 (0.01)	0.53 (0.01)

Table S3 *(continued)*

Chemical characteristics	Site C				Site D			
	Leaf litter		Fine roots		Leaf litter		Fine roots	
	Ambient	N deposition	Ambient	N deposition	Ambient	N deposition	Ambient	N deposition
Cell-wall fraction (%)	67.29 (5.79)	54.76 (1.90) (*)	84.10 (1.71)	86.24 (1.11)	64.67 (3.17)	73.72 (2.84)	83.42 (0.90)	83.32 (2.35)
AIF	14.49 (0.71)	13.57 (0.71)	45.90 (1.37)	46.72 (0.72)	14.81 (0.94)	14.81 (0.52)	46.42 (2.36)	45.16 (1.27)
Hemicellulose	13.67 (0.53)	13.04 (1.14)	15.71 (0.62)	15.82 (1.96)	13.96 (1.14)	14.99 (1.54)	16.09 (2.02)	15.64 (1.10)
Cellulose	39.13 (5.00)	28.15 (1.78)	22.49 (1.00)	23.70 (2.36)	35.90 (1.45)	43.92 (1.15)	20.91 (4.96)	22.53 (0.82)
Extractable fraction (%)	32.71 (5.79)	45.24 (1.90)(*)	15.90 (1.71)	13.76 (1.11)	35.33 (3.17)	26.28 (2.84)	16.58 (0.90)	16.68 (2.35)
Soluble phenolics	10.92 (2.32)	13.44 (1.09)	4.11 (0.73)	3.16 (0.28)	11.55 (1.48)	9.71 (0.87)	4.57 (0.47)	4.79 (0.96)
Condensed tannins	4.80 (1.27)	5.73 (0.63)	13.02 (2.54)	9.90 (1.50)	4.50 (0.70)	2.95 (0.93)	14.82 (1.02)	13.36 (2.42)
Extractable fraction	4.29 (1.28)	5.21 (0.66)	10.54 (2.72)	7.79 (1.35)	3.84 (0.72)	2.42 (0.80)	11.75 (1.12)	10.73 (2.34)
Bound fraction	0.52 (0.06)	0.51 (0.09)	2.48 (0.37)	2.11 (0.17)	0.66 (0.13)	0.53 (0.13)	3.07 (0.74)	2.62 (0.13)
NSCs	4.25 (0.68)	5.47 (1.29)	1.74 (0.07)	1.66 (0.29)	4.23 (0.61)	3.79 (0.38)	1.94 (0.23)	1.92 (0.52)
Lipids	7.05 (1.09)	7.56 (0.63)	3.07 (0.56)	3.31 (0.32)	8.24 (0.16)	8.23 (0.76)	3.89 (0.27)	3.60 (0.07)
Soluble proteins	1.06 (0.11)	1.19 (0.13)	3.58 (0.72)	2.80 (0.28)	1.03 (0.10)	0.87 (0.22)	3.12 (0.34)	3.08 (0.39)
Unidentified	9.42 (2.32)	17.58 (3.70)	3.40 (0.35)	2.83 (0.35)	10.28 (1.38)	4.10 (3.64)	3.07 (0.81)	3.30 (0.50)
N (%)	0.61 (0.07)	0.66 (0.05)	1.66 (0.05)	1.78 (0.04)	0.64 (0.04)	0.71 (0.07)	1.46 (0.15)	1.51 (0.12)
Litter quality indices (ratio)								
AIF : N	24.06 (4.04)	20.52 (2.36)	27.84 (1.61)	26.36 (0.27)	23.25 (1.39)	21.02 (1.19)	32.32 (4.78)	30.19 (3.20)
C : N	80.11 (6.51)	76.40 (6.56)	31.33 (1.01)	29.06 (0.63)	77.79 (6.12)	70.46 (5.32)	35.75 (3.99)	34.59 (2.98)
Lignocellulose index	0.22 (0.02)	0.25 (0.02)	0.55 (0.01)	0.54 (0.01)	0.23 (0.004)	0.20 (0.001)	0.56 (0.03)	0.54 (0.01)

Values are means (SD) for each treatment in three plots from each site (n = 3). Significant N effects at: (*), $P < 0.1$; *, $P < 0.05$; **, $P < 0.01$. AIF, acid-insoluble fraction; NSCs, non-structural carbohydrates.

Table S4 Major biochemical components and three litter quality indices of spring and autumn fine roots across the four forest sites receiving simulated nitrogen (N) deposition

Chemical characteristics	Spring		Autumn		Main effects
	Ambient	N deposition	Ambient	N deposition	
Cell-wall fraction (%)	84.5^{b} (1.0)	84.4^{b} (2.4)	82.6^{a} (1.8)	84.4^{b} (2.8)	Season, Site
AIF	45.8^{b} (2.4)	46.1^{b} (1.5)	44.3^{a} (2.2)	45.4^{a} (2.2)	Season
Hemicellulose	16.4^{a} (1.3)	15.9^{a} (1.5)	15.3^{b} (1.7)	15.5^{b} (1.4)	Season
Cellulose	22.3^{a} (3.0)	22.4^{a} (1.9)	23.0^{a} (2.9)	23.4^{a} (3.4)	
Extractable fraction (%)	15.5^{a} (1.0)	15.6^{a} (2.4)	17.4^{b} (1.8)	15.6^{a} (2.8)	Season, Site
Soluble phenolics	3.91^{a} (0.70)	3.82^{a} (1.29)	3.99^{a} (0.63)	3.66^{a} (0.75)	Site
Condensed tannins†	13.2^{a} (1.5)	12.2^{a} (2.1)	14.0^{a} (2.7)	12.5^{a} (3.9)	Site, N
NSCs	1.38^{a} (0.21)	1.45^{a} (0.44)	2.37^{b} (0.38)	2.24^{b} (0.39)	Season
Lipids	3.31^{ab} (0.53)	3.11^{a} (0.33)	3.89^{c} (0.71)	3.70^{bc} (0.48)	Season
Soluble proteins	3.12^{ab} (1.31)	2.86^{a} (0.50)	3.45^{b} (0.55)	3.03^{ab} (0.47)	Season, N
Unidentified‡	3.76^{b} (0.98)	4.37^{b} (0.87)	3.66^{ab} (1.24)	2.99^{a} (1.54)	Season, Site
N (%)	1.49^{a} (0.15)	1.58^{ab} (0.14)	1.61^{bc} (0.24)	1.70^{c} (0.04)	Season, Site, N
Litter quality indices (ratio)					
AIF : N	31.1^{b} (3.4)	29.5^{b} (2.9)	28.0^{ab} (4.1)	26.8^{a} (2.4)	Season, Site
C : N	33.9^{b} (3.0)	32.5^{ab} (3.5)	32.8^{ab} (5.34)	30.8^{a} (2.0)	Season, Site
Lignocellulose index	0.54^{a} (0.03)	0.55^{a} (0.01)	0.54^{a} (0.02)	0.54^{a} (0.03)	

Values are means (SD) with three replicated plots for each treatment at each of four sites (n = 12). Different letters in the same row indicate significant differences ($P < 0.05$). Significant main effects are shown ($P < 0.05$). AIF, acid-insoluble fraction; NSCs, non-structural carbohydrates. †Condensed tannins (CTs) are a subset of plant phenolics. There is no generally-accepted CT standard for the acid-butanol assays used to determine CTs. Thus, the CT concentrations reported here should be interpreted more as relative

comparisons between fine roots and leaf litter than absolute quantification. Bound tannins could be double-counted in AIF in this table, however, bound CTs only represented 11.8 % and 20.9 % of total CTs by average in fine roots and leaf litter, respectively (Table S3). ‡Unidentified portion is the difference between extractable fraction and the sum of soluble phenolics, non-structural carbohydrates, lipids, and soluble proteins.

Table S5 Analysis of two-way ANOVA (site × N deposition) on the annual litter production of leaf litter, fine roots, and total litter at the four northern hardwood forest study sites

Source of variation	df	Leaf litter		Fine roots		Total litter production	
		F	*P*	*F*	*P*	*F*	*P*
Study site	3	29.52	**<0.001**	19.59	**<0.001**	19.48	**<0.001**
N deposition	1	1.68	0.213	0.60	0.450	0.05	0.825
Site × N	3	0.69	0.573	1.41	0.276	1.24	0.326
Error	16						

Bold numbers: $P < 0.05$.

Table S6 Mixed linear model analysis of biochemical fluxes among study sites, nitrogen deposition treatments, and tissue types in a split-plot design

Chemical characteristics	Whole-plot						Within-plot							
	Site df = 3 / 16		N deposition df = 1 / 16		Site × N df = 3 / 16		Tissue df =1 / 16		Tissue × Site df =3 / 16		Tissue × N df =1 / 16		Tissue × Site × N df =3 / 16	
	F	*P*	*F*	*P*	*F*	*P*	*F*	*P*	*F*	*P*	*F*	*P*	*F*	*P*
AIF	15.43	**<0.001**	1.29	0.272	1.03	0.405	538.20	**<0.001**	19.09	**<0.001**	0.02	0.890	2.74	0.078
Hemicellulose	10.29	**<0.001**	0.19	0.669	0.88	0.473	3.26	0.090	9.35	**<0.001**	0.51	0.487	1.07	0.391
Cellulose	10.80	**<0.001**	2.07	0.169	3.38	**0.044**	356.97	**<0.001**	25.16	**<0.001**	0.86	0.368	2.39	0.107
Soluble phenolics	11.61	**<0.001**	0.27	0.610	1.22	0.333	557.3	**<0.001**	15.78	**<0.001**	2.42	0.139	2.61	0.087
Condensed tannins	3.62	**0.036**	5.27	**0.036**	0.27	0.848	80.47	**<0.001**	10.56	**<0.001**	0.25	0.627	3.65	**0.035**
NSCs	13.94	**<0.001**	0.25	0.627	1.21	0.338	504.86	**<0.001**	18.79	**<0.001**	4.24	0.056	1.44	0.268
Lipids	17.86	**<0.001**	0.59	0.455	0.07	0.977	387.13	**<0.001**	11.79	**<0.001**	1.61	0.223	0.81	0.507
Soluble proteins	4.90	**0.013**	4.32	0.054	0.20	0.894	224.78	**<0.001**	12.54	**<0.001**	0.54	0.473	3.98	**0.027**
Nitrogen	10.31	**<0.001**	7.59	**0.014**	3.21	0.051	179.52	**<0.001**	7.19	**0.003**	7.00	**0.018**	0.35	0.792

The mixed linear models included fixed effects shown in the table and random effects of plots nested (n = 3) in (site × N treatment). The degree of freedom is shown as 'numerator df / denominator df'. Bold numbers: $P < 0.05$. AIF, acid-insoluble fraction; NSCs, non-structural carbohydrates.

Table S7 Analysis two-way ANOVA (site × N deposition) on the combined fluxes of leaf litter and fine root biochemical fluxes at the four northern hardwood forest study sites

Sum fluxes	Study site		N deposition		Site × N	
	F	*P*	*F*	*P*	*F*	*P*
AIF	15.37	**<0.001**	0.41	0.529	1.37	0.287
Hemicellulose	10.05	**<0.001**	0.14	0.709	0.71	0.563
Cellulose	9.22	**<0.001**	2.99	0.103	5.16	**0.011**
Soluble Phenolics	1.19	0.346	0.43	0.522	0.99	0.421
Condensed tannins	11.78	**<0.001**	3.56	0.077	0.43	0.723
NSCs	3.61	**0.036**	3.65	0.074	1.54	0.242
Lipids	21.32	**<0.001**	0.04	0.841	0.04	0.988
Soluble proteins	10.20	**<0.001**	3.19	0.093	1.07	0.391
Nitrogen	13.30	**<0.001**	4.18	0.058	3.47	**0.041**

Bold numbers: $P < 0.05$. AIF, acid-insoluble fraction; NSCs, non-structural carbohydrates.

Methods S1 Sequential extraction for the extractive-free fraction

Extractive-free fraction was obtained with a sequential extraction procedure (Friend, 1992; Booker *et al.,* 1996). Extractive-free tissue was prepared by washing 25 mg samples with 50% methanol (3×), methanol : chloroform : water (2.0 : 1.0 : 0. 8) (3×), phenol : acetic acid : water (PAW, 2.0 : 1.0 : 0. 9) (3×, with an overnight incubation during the second wash), and ethanol (5×), with centrifugations (1400 ***g***, 5 min) between washings. PAW washes were used to remove bulk proteins from the residue (Laird *et al.,* 1976; Friend, 1992). Samples were dried at 70°C and weighed as the mass of extractive-free fraction.

References

Booker FL, Anttonen S, Heagle AS. 1996. Catechin, proanthocyanidin, and lignin contents of loblolly pine (Pinus taeda) needles after chronic exposure to ozone. *New Phytologist* **132**: 483–492.

Friend J. 1992. Lignin and associated phenolic acids in cell walls. In: Gurr SJ, McPherson MJ, eds. *Molecular plant pathology: a practical approach*. Oxford, UK: IRL Press.

Laird WM, Mbadiwe EI, Synge RL. 1976. A simplified procedure for fractionating plant materials. *Journal of the Science of Food and Agriculture* **27**: 127–130.
